# Recent Advances of Pullulan and/or Dextran-Based Materials for Bone Tissue Engineering Strategies in Preclinical Studies: A Systematic Review

**DOI:** 10.3389/fbioe.2022.889481

**Published:** 2022-06-30

**Authors:** Naïma Ahmed Omar, Joëlle Amédée, Didier Letourneur, Jean-Christophe Fricain, Mathilde Fenelon

**Affiliations:** ^1^ Université de Bordeaux, INSERM U1026, BIOTIS, Bordeaux, France; ^2^ SILTISS, Saint-Viance, France; ^3^ Université Paris Cité, Université Sorbonne Paris Nord, INSERM U1148, LVTS, X Bichat Hospital, Université de Paris, Paris, France; ^4^ Service de Chirurgie Orale, CHU Bordeaux, Bordeaux, France

**Keywords:** pullulan, dextran, bone tissue engineering, natural polymers, polysaccharides, bone regeneration

## Abstract

Bone tissue engineering (BTE) strategies are increasingly investigated to overcome the limitations of currently used bone substitutes and to improve the bone regeneration process. Among the natural polymers used for tissue engineering, dextran and pullulan appear as natural hydrophilic polysaccharides that became promising biomaterials for BTE. This systematic review aimed to present the different published applications of pullulan and dextran-based biomaterials for BTE. An electronic search in Pubmed, Scopus, and Web of Science databases was conducted. Selection of articles was performed following PRISMA guidelines. This systematic review led to the inclusion of 28 articles on the use of pullulan and/or dextran-based biomaterials to promote bone regeneration in preclinical models. Sixteen studies focused on dextran-based materials for bone regeneration, six on pullulan substitutes and six on the combination of pullulan and dextran. Several strategies have been developed to provide bone regeneration capacity, mainly through their fabrication processes (functionalization methods, cross-linking process), or the addition of bioactive elements. We have summarized here the strategies employed to use the polysaccharide scaffolds (fabrication process, composition, application usages, route of administration), and we highlighted their relevance and limitations for BTE applications.

## 1 Introduction

Improving bone regeneration after traumatic injuries, pathologies, or tumors resection has been and still remains a surgical challenge since decades. To date, autologous bone grafting (Dimitriou et al., 2011; Lanza et al., 2014) is the gold standard technique for bone reconstruction, as it is induced by a non-immunogenic way, the properties required for bone regeneration [i.e., osteoinduction, osteoconduction, and osteogenesis (Dimitriou et al., 2011; Lanza et al., 2014)]. However, this method has some important drawbacks due to its limited availability and inherent complications from the surgical procedures (donor site injury and morbidity) (Dimitriou et al., 2011).

Bone tissue engineering (BTE) strategies have thus emerged to develop alternatives to conventional autologous bone graft reconstruction. BTE approach is usually based on a combination of scaffolds with cells and/or bioactive molecules to provide the conditions required for bone formation. Design of scaffolds that mimic the mechanical and structural features of bone extracellular matrix (Filippi et al., 2020) that is highlighted. Biomaterials for bone scaffolding applications are critical to enable cell viability and proliferation, osteodifferentiation, angiogenesis, host integration, and when needed load bearing (Roseti et al., 2017; Kashirina et al., 2019). Calcium phosphate cements (CPC) such as hydroxyapatite (HA) or β-tricalcium phosphate (β-TCP) are thus the most commonly used scaffolds for BTE applications (Ana et al., 2018; Liang et al., 2021).

Recently, a number of natural polymers, such as chitosan, collagen, gelatin, hyaluronic acid, or alginate has gained an increasing interest (Tang et al., 2021). They often show biocompatibility, biodegradability, and share similar structures to the natural extracellular matrix (Filippi et al., 2020). An interesting property of polymeric scaffolds is their ability to be easily designed according to the desired three-dimensional structure, such as hydrogels, macroporous scaffolds, microspheres, or micro-molded matrices (Mallick and Cox, 2013; Wu et al., 2021). Polysaccharides are one type of natural polymers that are composed of molecules linked through a glycosidic linkage (Hussain et al., 2017). Among them, dextran and pullulan are both exopolysaccharides, which are secreted into the surrounding environment of microorganisms by cell wall-anchored enzymes (Hussain et al., 2017). They have already been used for medical research since they are biocompatible, biodegradable, and present no immunogenic reaction (Prajapati et al., 2013; Banerjee and Bandopadhyay, 2016). For example, dextran was used as plasma expander (Banerjee and Bandopadhyay, 2016), drug carrier to target organs [e.g., colon (Hovgaard and Brøndsted, 1995; Simonsen et al., 1995), skin (Sun et al., 2011)] or as a molecular imaging tracer for magnetic resonance imaging (Tassa et al., 2011; Banerjee and Bandopadhyay, 2016) (e.g., dextran-coated iron oxide nanoparticles). Pullulan-based materials have been used as an excipient in pharmaceutical tablets (Prajapati et al., 2013). Some researchers also focused on its potential as a plasma-blood substitute (Prajapati et al., 2013) like dextran derivatives, as a drug delivery system (Fundueanu et al., 2003) or as a fluorescent probe for medical imaging (Morimoto et al., 2005b).

The use of these two polysaccharides for BTE has thus been investigated to design biological scaffolds for bone regeneration. In the last decades, several studies focused on the production of pullulan and/or dextran-based scaffolds combined with CPC or growth factors, and investigated their potential for BTE. However, there is no report summarizing the development of pullulan and/or dextran-based bone materials, functionalization methods and their applications to promote bone regeneration. This review aims to identify the different strategies of pullulan and/or dextran-based substitutes for bone tissue engineering applications in preclinical studies since no clinical application were reported in the online search.

## 2 Materials and Methods

This systematic review was performed according to the Preferred Reporting Items for Systematic Reviews and Meta-Analyses (PRISMA) guidelines (Moher et al., 2015). A protocol was specified and registered on the database International Prospective Register of Systematic Reviews (PROSPERO) (registration number CRD42021220920) and is available from: https://www.crd.york.ac.uk/prospero/display_record.php?ID=CRD42021220920.

### 2.1 Focused Question

This systematic review was performed to address the following focused question: “What are the best strategies of using pullulan and/or dextran in bone regeneration in preclinical models?”

### 2.2 Search Strategy

An electronic search of the MedLine (PubMed), Embase (Scopus), and Web of science databases was carried out. Medical subject headlings (MeSH) terms were combined with keywords and Boolean operators to search databases. All searches were performed from October 2020 to February 2022 by focusing on studies written in English or French and published between January 2000 and February 2022. The searching query used for the research was: (“pullulan” OR “dextran”) AND [“bone (MeSH)” OR “bone regeneration (MeSH)”]. Additional articles were also searched by manually screening the list of references of all publications selected by the search.

### 2.3 Eligibility Criteria

Preclinical controlled trials using pullulan and/or dextran substitutes to induce bone regeneration were considered. All animal studies (all type, all sexes) were eligible if they assessed new bone formation in a bone defect or subcutaneously. *In vitro* studies, clinical trials and reviews were excluded.

### 2.4 Screening Methods and Data Extraction

Two independent reviewers (NA and MF) screened the titles and abstracts. For eligible studies that matched with the inclusion criteria, full texts were then assessed. Any disagreement between the reviewers over the eligibility of particular studies was resolved through discussion with a third reviewer (J-CF).

In order to extract relevant data from included studies, structurable tables were made with the following data: authors, animal models, type of defect or implantation site, conditions tested (with the number of defects created or scaffolds implanted), composition of the scaffolds, cross-linking reagents, adjuvants (e.g., growths factors, cell lines, mineralized molecules), material design, observation period, and experimental analysis with results. For any missing data, authors were contacted by e-mail to complete tables.

### 2.5 Quality Assessment and Analysis of the Data

Methodological quality of individual studies was assessed using the collaborative Evidence-Based Complementary and Alternative Medicine approach to meta-analysis and review of animal data in experimental infarction (CAMARADES) 10-item quality checklist (Macleod et al., 2004) and SYRCLE’s risk of bias tool (Hooijmans et al., 2014). These tools enabled to build a modified checklist by using eight items for relevance: 1) Peer reviewed publication; 2) Control of the temperature in the animal facilities; 3) Random allocation to treatment or control; 4) Blinded assessment of outcomes; 5) Animal model description; 6) Sample size calculation; 7) Compliance with animal welfare regulation; 8) Statement of potential conflict of interest. Data analysis was then performed in a descriptive way since the information obtained did not enable meta-analyses.

## 3 Results

### 3.1 Search Outcomes

**FIGURE 1:**
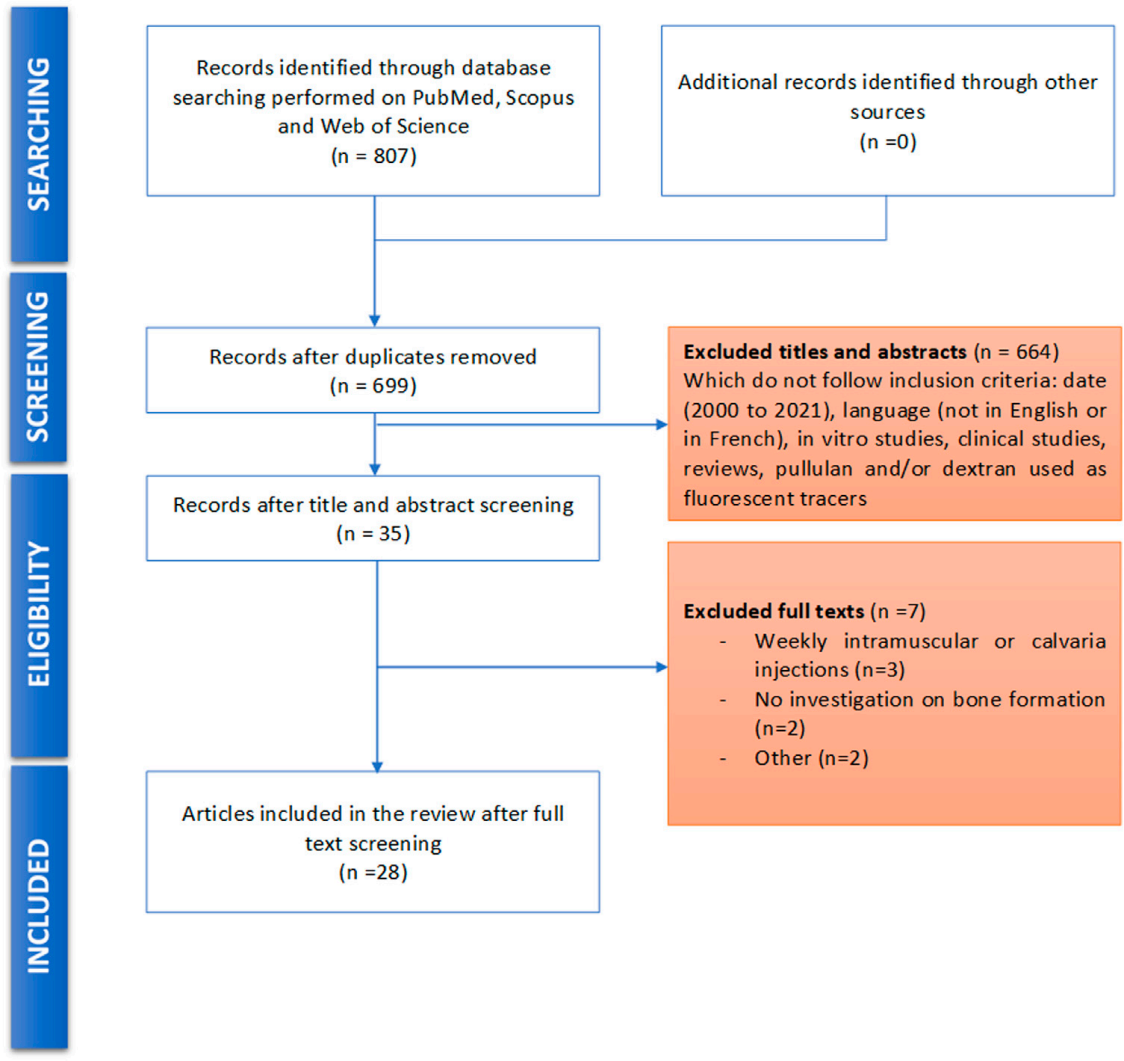
Flow diagram of the screened publications.

**FIGURE 2:**
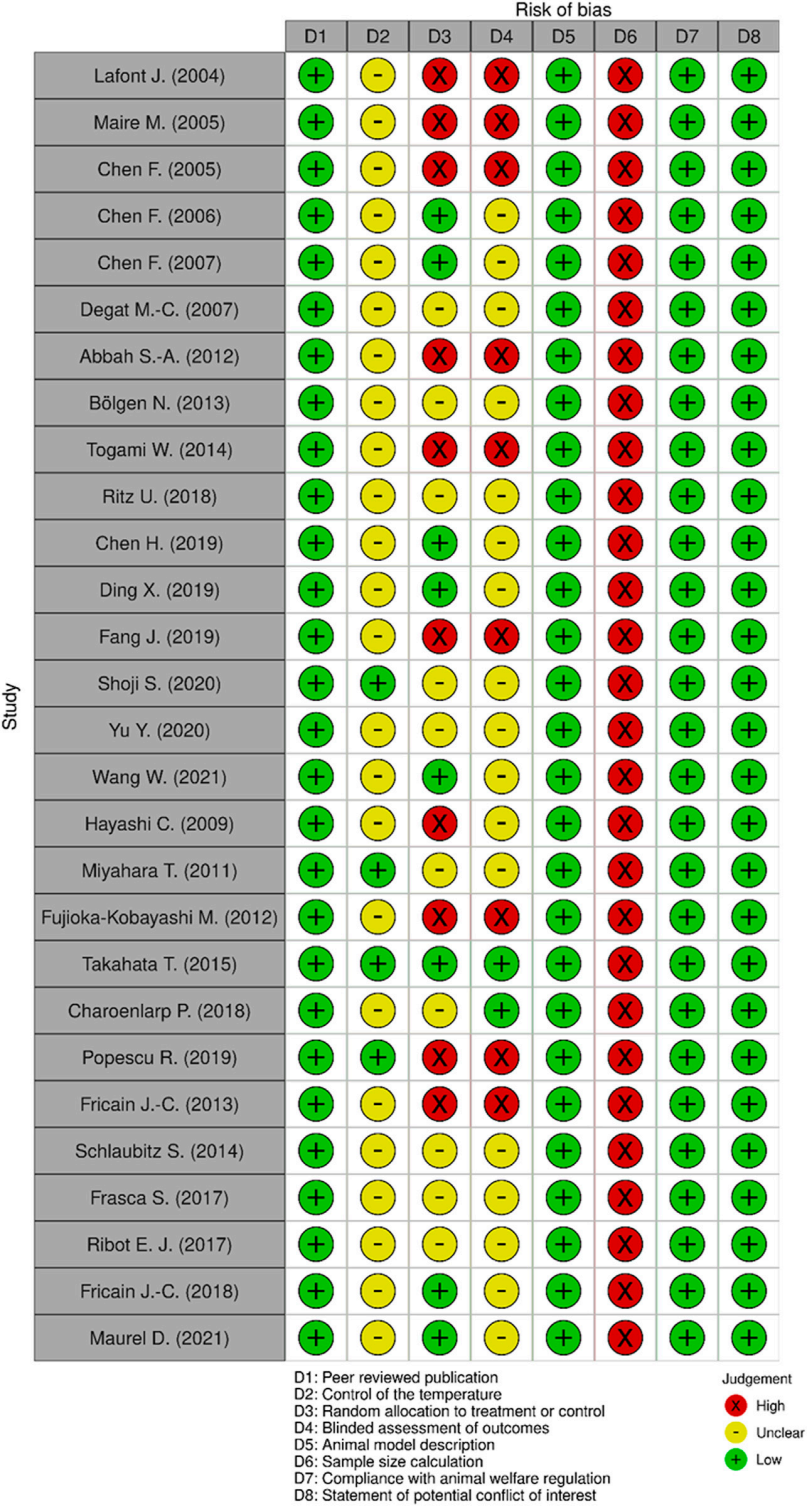
Quality assessment of each included study using a modified CAMARADES checklist.

**FIGURE 3:**
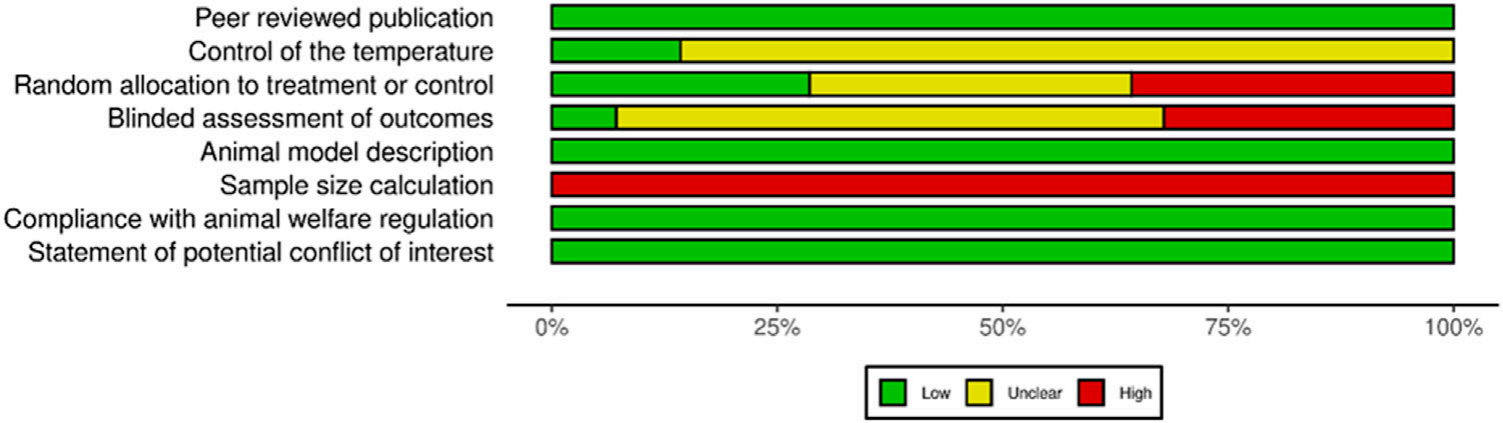
Summary of risk of bias assessment for the included studies using a modified CAMARADES checklist.

Database screening yielded to 713 publications after duplicates removal. Among these publications, 35 articles were selected for potential inclusion after title and abstract reading. Full texts of these 35 articles were reviewed. Seven of the 35 studies were excluded for not meeting the inclusion criteria. No additional study was added for selection after manually screening the list of references of selected publications. Finally, 28 studies were included for this systematic review: 16 used dextran-derived biomaterials (Lafont et al., 2004; Maire et al., 2005a; Chen et al., 2005; Chen et al., 2006; Chen et al., 2007; Degat et al., 2009; Abbah et al., 2012; Bölgen et al., 2014; Ritz et al., 2018; Chen et al., 2019; Ding et al., 2019; Fang et al., 2019) for bone regeneration, six used pullulan-derived biomaterials (Hayashi et al., 2009; Fujioka-Kobayashi et al., 2012; Miyahara et al., 2012; Takahata et al., 2015; Charoenlarp et al., 2018; Popescu et al., 2020) and six used a combination of these two polysaccharides (Fricain et al., 2013; Fricain et al., 2018; Schlaubitz et al., 2014; Frasca et al., 2017; Ribot et al., 2017) (Table 1). A flowchart of the selection and inclusion process, based on PRISMA recommendations is presented in Figure 1. Risk of bias ranged from low to high and detailed results of methodological quality are presented in Figures 2, 3. Sample size calculation and blinded assessment of outcomes showed the highest risk of bias.

**TABLE 1:** Characterization of scaffold used in the included studies.

Author (Year)	Polysaccharide composition	Biocomposite scaffold (additional polymers and ceramics)	Functionalization of dextran, pullulan or pullulan/dextran scaffolds	Cross-linking process (cross-linker used)	Material aspect	Bioactive components
Lafont et al. (2004)	Dextran	—	Carboxymethyl, benzylamide and sulfate	—	Aqueous solution (loaded into collagen sponges)	—
Maire et al. (2005a)	Dextran	—	Carboxylate, benzylamide and sulfate (three different D.S. expressed in %: 0, 2, and 18)	Chemical cross-linking (STMP)	Hydrogel	BMP-2
Chen et al. (2005)	Dextran	Gelatin	—	Chemical cross-linking	Microspheres	BMP-2
Chen et al. (2006)	Dextran	Gelatin	Glycidyl methacrylate (three different D.S. referring to the number of methacrylated groups per 100 glucopyranose residues: 4.7, 6.3, and 7.8)	Chemical cross-linking (Polyethylene glycol)	Microspheres	IGF-1
Chen et al. (2007)	Dextran	Gelatin	Glycidyl methacrylate	Chemical cross-linking	Hydrogel	—
	Dextran	Polyethylene glycol	Glycidyl methacrylate	Chemical cross-linking	Microspheres	BMP-2
Degat et al. (2007)	Dextran	—	Carboxymethyl, benzylamide	—	Aqueous solution (loaded into collagen sponges)	BMP-2
Abbah et al. (2012)	Dextran/Alginate	—	Diethylaminoethyl	Physical cross-linking (CaCl_2_)	Microspheres (loaded into polymeric sponges)	BMP-2
Bölgen et al. (2013)	Dextran	Hydroxyethyl methacrylate	—	Chemical cross-linking (Methylenebisacrylamide)	Cryogel (disc-shaped)	MSCs
		Lactate				
Togami et al. (2014)	Dextran	Polyvinyl formal	—	—	Sponges	—
Ritz et al. (2018)	Dextran	—	Carboxymethyl and epoxy benzophenone	Chemical cross-linking (UV radiation)	Hydrogel (disc-shaped)	BMP-7
						SDF-1
						HUVEC hOB
Chen et al. (2019)	Dextran	—	Sulfate	—	Aqueous solution (loaded into gelatin sponges)	BMP-2
Ding et al. (2019)	Dextran/Chitosan	Strontium-doped HA	Formylbenzoic	Chemical cross-linking	Hydrogel (injected, *in situ* forming)	—
Fang et al. (2019)	Dextran	Polyacrylamide	Urethane methacrylate	Physical cross-linking (SDS/SMA micelles)	Hydrogel (disc-shaped)	—
		HA				
Shoji et al. (2020)	Dextran	—	Tyramine	Chemical cross-linking (H_2_O_2_, HRP)	Hydrogel (injected, *in situ* forming)	bFGF
Yu et al. (2020)	Dextran	—	—	—	Aqueous solution (loaded into gelatin sponges)	BMP-2
Wang et al. (2021)	Dextran	PLGA	Aldehyde and catechol	Chemical cross-linking (Schiff reaction)	Hydrogel (injected, *in situ forming*)	Bisphosphonate
		HA				
Hayashi et al. (2009)	Pullulan	—	Cholesteryl and acryloyl	Chemical cross-linking (thiol-bearing polyethylene glycol)	Hydrogel (hemisphere-shaped)	BMP-2
Miyahara et al. (2011)	Pullulan	—	Cholesteryl and acryloyl	Chemical cross-linking (thiol-bearing polyethylene glycol)	Hydrogel (membrane)	—
Fujioka-Kobayashi et al. (2012)	Pullulan	—	Cholesteryl and acryloyl	Chemical cross-linking (thiol-bearing polyethylene glycol)	Hydrogel (disc-shaped)	BMP-2 and/or FGF18
Takahata et al. (2015)	Pullulan	β-TCP	Phosphate	—	NS	—
Charoenlarp et al. (2018)	Pullulan	—	Cholesteryl and acryloyl	Chemical cross-linking (thiol-bearing polyethylene glycol and/or RGD peptides)	Hydrogel (disc-shaped)	BMP-2 and FGF18
Popescu et al. (2019)	Pullulan/Alginate	Bioglass containing X%CuO (X = 0.5 or 1.5)	—	—	NS	—
Fricain et al. (2013)	Pullulan/Dextran	HA	—	Chemical cross-linking (STMP)	Hydrogel (sponges)	—
Schlaubitz et al. (2014)	Pullulan/Dextran	HA	—	Chemical cross-linking (STMP)	Microspheres	—
Frasca et al. (2017)	Pullulan/Dextran	—	—	Chemical cross-linking (STMP)	Hydrogel (sponges)	MSCs
Ribot et al. (2017)	Pullulan/Dextran	HA and/or fucoidan	—	Chemical cross-linking (STMP)	Microspheres	—
Fricain et al. (2018)	Pullulan/Dextran	HA	—	Chemical cross-linking (STMP)	Microspheres	—
Maurel et al. (2021)	Pullulan/Dextran	HA	—	Chemical cross-linking (STMP)	Microspheres	—

bFGF, basic fibroblast growth factor; BMP, bone morphogenetic protein; β-TCP, beta-tricalcium phosphate; CuO, copper oxide; D.S., degree of substitution; FGF, fibroblast growth factor; HA, hydroxyapatite; HEMA, hydroxyethyl methacrylate; H_2_O_2,_ hydrogen peroxide; hOB, human osteoblast; HRP, horseradish peroxidase; HUVEC, human umbilical vein endothelial cells; IGF, insulin-like growth factor; MSCs, mesenchymal stromal cells; NS, not specified; PEG, polyethylene glycol; PLGA, poly-(L-glutamic acid); RGD, arginine-glycine-aspartate; SDF-1, stromal-derived growth factor; SDS, sodium dodecyl sulfate; SMA, stearyl methacrylate; STMP, sodium trimetaphosphate; (—), not applicable.

### 3.2 Experimental Models

**FIGURE 4:**
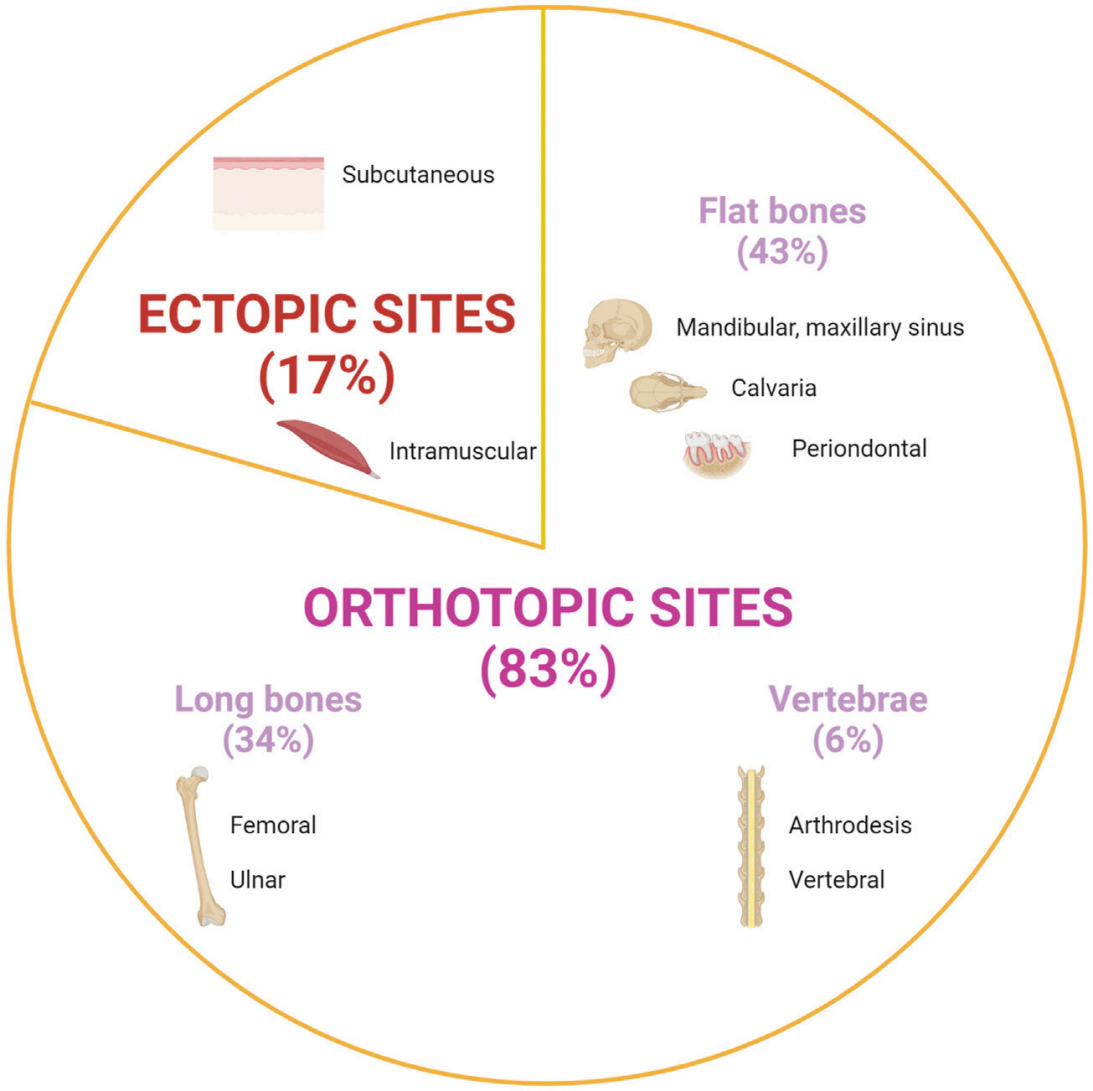
Implantation sites of pullulan and/or dextran-based scaffolds to assess their potential for bone regeneration. Created with BioRender.com.

Most studies (25 out of 28 studies) were carried out on small animals (e.g., mice, rats, rabbits), and only few studies investigated bone formation in larger animal models (e.g., pigs, sheeps, goats, and dogs). Experimental procedures were performed ectopically (17%), or orthotopically (83%) (Figure 4). Ectopic bone formation was investigated in six studies using subcutaneous implantation in the back of animals or by muscular implantation (e.g., leg muscles) (Table 2) (Maire et al., 2005a; Degat et al., 2009; Hayashi et al., 2009; Fricain et al., 2013; Chen et al., 2019; Yu et al., 2020). Orthotopic implantations were mainly performed on maxillofacial defects and long bone defects (Tables 3, 4, 5) (Lafont et al., 2004; Chen et al., 2005; Chen et al., 2006; Chen et al., 2007; Hayashi et al., 2009; Abbah et al., 2012; Fujioka-Kobayashi et al., 2012; Miyahara et al., 2012; Fricain et al., 2013; Bölgen et al., 2014; Schlaubitz et al., 2014; Takahata et al., 2015; Togami et al., 2015; Frasca et al., 2017; Ribot et al., 2017; Charoenlarp et al., 2018; Fricain et al., 2018; Ritz et al., 2018; Ding et al., 2019; Fang et al., 2019; Popescu et al., 2020; Shoji et al., 2020; Maurel et al., 2021; Wang et al., 2021). Nine studies investigated calvarial defects, six studies focused on mandibular or maxillary defects (e.g., periodontal, sinus bone augmentation), whereas 10 studies were performed on femoral defects (e.g., condyle, epiphysis, metaphysis), one study on ulnar defect and one study on tibial defect. Two studies assessed vertebral bone defects regeneration. Bone formation was investigated radiographically mainly using micro-computed tomography (micro-CT) and/or by histological analysis.

**TABLE 2:** Preclinical studies using Dextran and/or Pullulan scaffold in ectopic sites.

Author (Year)	Animal model (sex; species); n, number of animals used	Implantation site, dimension of the implanted material	Conditions (N, number of total implanted materials per condition)	Observation time [in day (D), week (W) or month (M)]	Experimental analysis	Results
Maire et al. (2005b)	Rat (male, Sprague Dawley), *n* = 21	Muscle implantation (back)	1: BMP (N = 20)	7 W	2D X-ray (qualitative analysis)	Dextran (DS 18%) + BMP showed large radiopaque areas compared to other groups (no statistical significance due to heterogeneity within the same group)
			2: Dextran (DS 0%) + BMP (N = 14)			
		Collagen sponge Diameter: 7 mm Height: 7 mm	3: Dextran (DS 2%) + BMP (N = 14)		Stevenel blue and Van Gieson Picrofuschin staining (Bone formation); Von Kossa staining (qualitative analysis)	For Dextran (DS 18%) + BMP group, bone formation occurred through endochondral ossification or by intramembranous ossification
			4: Dextran (DS 18%) + BMP (N = 14)			
Degat et al. (2007)	Rat (male, Sprague Dawley), *n* = 24	Subcutaneous (back)	1: BMP-2 (N = 6)	4 W	Calcium mass (µg/implant)	Dextran (10 µg) + BMP-2 ** > BMP-2
			2: Dextran (1 µg) + BMP-2 (N = 6)			
		Collagen sponge	3: Dextran (10 µg) + BMP-2 (N = 6)			BMP-2 * > Dextran (100 µg) + BMP-2
		Diameter: 3 mm Height: 2 mm	4: Dextran (100 µg) + BMP-2 (N = 6)			
Chen et al. (2019)	Mouse (male, C57BL/6), *n* = 3	Muscle implantation (leg)	1: BMP-2 (N=NS)	2 W	Wet and ash weights of ectopic bone (g)	No significant difference observed between Dextran + BMP-2 and BMP-2 alone groups
		Gelatin sponge size	2: Chitosan + BMP-2 (N=NS)			
		Length: 0.5 cm	3: Dextran + BMP-2 (N=NS)			
		Width: 0.5 cm	4: PSS + BMP-2 (N=NS)	4 W	Masson’s Trichrome staining (qualitative analysis)	Dextran + BMP-2 less bone formation compared to other groups
		Height: 0.3 cm				
Yu et al. (2020)	Mouse (male, C56BL/6), *n* = 40	Muscle implantation (leg)	1: BMP-2 (10 µg) (N = 16)	2 W	Micro-CT (BVF in %)	No significant difference between groups
			2: BMP-2 (15 µg) (N = 16)			
		Gelatin sponge size: NS	3: Heparin + BMP-2 (N = 16)			
			4: Chitosan + BMP-2 (N = 16)	4 W	Masson’s Trichrome staining; TRAP+ staining (qualitative analysis)	At 4W: for Dextran + BMP-2 group, traces of trabecular bone and bone resorption occurred
			5: Dextran + BMP-2 (N = 16)			
Hayashi et al. (2009)	Mouse (male, ICR), *n* = NS	Muscle implantation (leg)	1: Pullulan (N = 3–4)	3 W	Micro-CT (BV in mm^3^)	No significant difference between Pullulan and Pullulan + BMP-2 group
		Diameter: 2.5 mm	2: Pullulan + BMP-2 (2 µg) (N = 3–4)			
		Height: NS				
Fricain et al. (2013)	Mouse (NS, Balb/c), *n* = NS	Subcutaneous (back)	1: Pullulan/Dextran (N = 6)	15 D	Micro-CT (MC in mg; MD in mg/cm^3^)	Pullulan/Dextran + HA ** > Pullulan/Dextran
		Diameter: 4 mm	2: Pullulan/Dextran + HA (N = 6)	30 D		
		Height: 4 mm		60 D	Enzyme Immunoassay for BMP-2 (pg/mg protein)	Pullulan/Dextran + HA * > Pullulan/Dextran
	Goat (NS) *n* = 7	Muscle implantation (back)	1: Pullulan/Dextran (N = 12)	1 M	Micro-CT (qualitative analysis)	Osteoid tissue formation for Pullulan/Dextran + HA group
		Diameter: 10 mm	2: Pullulan/Dextran + HA (N = 12)	6 M	Von Kossa staining; Masson’s Trichrome staining (qualitative analysis)	Osteoid tissue formation for Pullulan/Dextran + HA group
		Depth: 10 mm				

BMP, bone morphogenetic protein; BV, bone volume; BVF, bone volume fraction; D.S., degree of substitution; HA, hydroxyapatite; MC, mineral content; MD, mineral density; NS, not specified; PSS, poly (sodium-p-styrenesulfonate); * *p* < 0.05; ** *p* < 0.01.

**TABLE 3:** Preclinical studies using Dextran scaffolds for bone regeneration in orthotopic sites.

Author (Year)	Animal model (sex; species); n, number of animals used	Type of defect (defect size)	Conditions (N, number of defects created in total per condition)	Observation time [in day (D), week (W) or month (M)]	Experimental analysis	Results
Lafont et al. (2004)	Rat (male, Wistar), *n* = 98	Calvaria	1: PBS (N = 49)	1 D	2D x-ray analysis (Bone Repair in %)	At 7D: Dextran *** > PBS
				2 D		
				3 D		
				4 D		
		Ø: 5 mm	2: Dextran (N = 49)	5 D		
				6 D		
				7 D	Toluidine blue staining (ALP staining, qualitative analysis)	For Dextran group: at 5D, bone formation began at the edges of the defect and bone nodules appeared at 6D
Chen et al. (2005)	Dog (female, Beagle), *n* = 12	Periodontal class III furcation (2nd, 3rd and 4th premolars) H: 5 mm	1: CPC covered by Simple Membrane (N = 33)	8 W	HE staining (New bone area in %)	Dextran/Gelatin + BMP-2 ** > CPC
			2: CPC + BMP-2 covered by Simple Membrane (N = 32)			
			3: Dextran/Gelatin + BMP-2 in CPC covered by Functionalized Membrane (N = 34)			
Chen et al. (2006)	Dog (male, Beagle), *n* = 12	Periodontal class III furcation (2nd and 3rd molars)	1: Empty (N = 16)	4 W	2D X-Ray (qualitative analysis)	For Dextran/Gelatin containing IGF groups, new bone formation was observable
			2: Dextran (D.S. = 6.3)/Gelatin (N = 16)			
			3: IGF-1 (N = 16)			
		H: 5 mm	4: Dextran (D.S. = 4.7)/Gelatin + IGF-1 (N = 16)	8 W	HE staining; Modified Mallory’s Trichrome staining (Morphometric analysis of new bone in %)	Dextran (D.S. = 4.7)/Gelatin + IGF-1 * > Dextran (D.S. = 7.8)/Gelatin + IGF-1
			5: Dextran (D.S. = 6.3)/Gelatin + IGF-1 (N = 16)			
			6: Dextran (D.S. = 7.8)/Gelatin + IGF-1 (N = 16)			
Chen et al. (2007)	Dog (male, Mongrel), *n* = 6	Periodontal class III furcation (2nd and 3rd premolars)	1: Dextran/Gelatin (N = 16)	8 W	Modified Mallory’s Trichrome staining (Height of new bone regenerated in mm; % of regenerated new bone)	Dextran/Gelatin + microsphere BMP ** > Dextran/Gelatin + aqueous BMP * > Dextran/Gelatin
		H: 5 mm	2: Dextran/Gelatin + microsphere BMP (N = 16)			
			3: Dextran/Gelatin + aqueous BMP (N = 16)			
Abbah et al. (2012)	Rat (male, Sprague Dawley), *n* = 38	Arthrodesis (iliac bone L3, L4 fusion)	1: Empty (N = 6)	6 W	Micro-CT (BVF in %)	PLA + BMP-2 ** > DEAD-Dextran + BMP-2
		Bioresorbable mPCL-TCP scaffold	2: Alginate + BMP-2 (N = 8)			
		L: 4 mm	3: PLO + BMP-2 (N = 8)			
		W: 4 mm	4: PLA + BMP-2 (N = 8)			
		H: 4 mm	5: DEAE-Dextran + BMP-2 (N = 8)		Masson’s Trichrome staining (qualitative analysis)	DEAD-Dextran: new bone tissue was thin and sparse
Bölgen et al. (2013)	Rat (NS, Sprague Dawley), *n* = 57	Calvaria	1: HEMA/Lactate/Dextran (N = 29)	30 D	HE staining;	At 180D: HEMA/Lactate/Dextran > HEMA/Lactate/Dextran + MSCs *
		Ø: 8 mm	2: HEMA/Lactate/Dextran + MSCs (N = 28)	90 D	Masson’s Trichrome staining (New bone/Total cavity ratio)	
				180 D		
Togami et al. (2014)	Rabbit (male, Japanese) *n* = 9	Femoral (epiphysis)	1: PVF/Dextran without water holding capability (N = 9)	2 W	Micro-CT (BV/TV in %; BMD in mg/cm^3^)	At 4W and 6W: PVF/Dextran water holding capability ** > PVF/Dextran without water holding capability
		Ø: 4 mm	2: PVF/Dextran with water holding capability (N = 9)	4 W	HE staining (Ratio of bone formation per area in %)	PVF/Dextran water holding capability > PVF/Dextran without water holding capability ***
		H: 10 mm		6 W		Newly trabecular bone with lamellar structures for PVF/Dextran water holding capability
Ritz et al. (2018)	Mouse (NS, Athymic nude), *n* = 40	Calvaria	1: Empty (N = 2)	8 W	Micro-CT (BV/TV in Voxel)	Dextran + HUVEC * > Empty
		Ø: 2.7 mm	2: Dextran (N = 16)			Dextran + SDF-1 * > Empty
			3: Dextran + HUVEC (N = 16)			
			4: Dextran + HUVEC/hOB (N = 14)		HE staining (qualitative analysis)	For Dextran + HUVEC or SDF-1 groups: fibrous membrane structure complemented by a thin bony structure covering the whole defect
			5: Dextran + SDF-1 (N = 16)			
			6: Dextran + BMP-7 (N = 16)			
Ding et al. (2019)	Rat (NS, Sprague Dawley), *n* = 24	Calvaria	1: Chitosan/Dextran (N = 6)		Micro-CT (BV/TV in %)	Chitosan/Dextran + Sr100HA* > Chitosan/Dextran + HA * > Chitosan/Dextran
			2: Chitosan/Dextran + HA (N = 6)	4 W		
		Ø: 5 mm	3: Chitosan/Dextran + Sr50HA (N = 6)	8 W	HE staining (New bone area fraction in %); Masson’s Trichrome staining (Regenerated collagen in %)	Chitosan/Dextran + Sr100HA * > Chitosan/Dextran
			4: Chitosan/Dextran + Sr100HA (N = 6)			
Fang et al. (2019)	Rabbit (female, New Zealand), *n* = 18	Femoral (condyle)	1: Dextran/Polyacrylamide (N = 18)	0 D	Micro-CT (BMC in mg; BV in mm^3^)	At 30D: HA-Dextran/Polyacrylamide ** > Dextran/Polyacrylamide
		Ø: 3 mm	2: Dextran/Polyacrylamide + HA (N = 18)	30 D		
		H: 5 mm		90 D	HE staining; Masson’s Trichrome staining (qualitative analysis)	At 30D: HA-Dextran/Polyacrylamide groups sowed osteoid tissue formation which was not observed without HA
Shoji et al. (2020)	Mouse (NS, C57BL/6J), *n* = NS	Femur fracture	1: Empty (N=NS)	4 W	Micro-CT (BV in mm^3^; BMC in mg)	Dextran + bFGF * > Empty
		L: 10 mm	2: Dextran + PBS (N = 8)			
		W: 4 mm	3: Dextran + bFGF (N = 8)		HE staining (qualitative analysis)	Large calluses and newly formed bone at fracture site for Dextran + bFGF group
Wang et al. (2021)	Rats (female, Sprague Dawley) *n* = 18	Calvaria	1: Empty (N = 9)	4 W	Micro-CT (BVF in %; BMD in g/cc; trabecular number in mm^−1^)	Dextran/PLGA + HA + BP * > Empty
		Ø: 4 mm	2: Dextran/PLGA + HA + BP (N = 9)	8 W		
				12 W	HE staining; Toluidine blue staining (qualitative analysis)	At 8W: for Dextran/PLGA + HA + BP group, large area of woven bone and thin lamellar structure

BMC, bone mineral content; BMD, bone mineral density; BMP, bone morphogenic protein; BP, bisphosphonate; BV, bone volume; BV/TV, bone volume over total volume; β-TCP, beta-tricalcium phosphate; BVF, bone volume fraction; CPC, calcium phosphate ceramic; DEAE, diethylaminoethyl; D.S., degree of substitution; H, height; HA, hydroxyapatite; HE, hematoxylin and eosin; HEMA, hydroxyethyl methacrylate; hOB, human osteoblast; HUVEC, human umbilical vein endothelial cells; L, length; MC, mineral content; MD, mineral density; Micro-CT, micro-computed tomography; mPCL-TCP, medical grade poly (ε-caprolactone)—β-tricalcium phosphate; MSCs, mesenchymal stromal cells; n/s not significant; NaCl, sodium chloride; PBS, phosphate buffer saline; PLA, poly-L-arginine; PLO, poly-L-ornithine; PVF, polyvinyl formol; Sr, strontium; W, width * *p* < 0.05; ** *p* < 0.01; *** *p* < 0.001; ø, diameter.

**TABLE 4:** Preclinical studies using Pullulan scaffolds for bone regeneration in orthotopic sites.

Author (Year)	Animal model (sex; species); n, number of animals used	Type of defect (defect size)	Conditions (N, number of defects created in total per condition)	Observation time [in day (D), week (W) or month (M)]	Experimental analysis	Results
Hayashi et al. (2009)	Mouse (male, ICR), *n* = NS	Calvaria implantation (without defect)	1: Pullulan (N=NS)	14 D	Micro-CT (BV in mm^3^)	No significant difference between groups
			2: Pullulan + BMP-2 (0.1 µg) (N=NS)			
		Ø: 4.6 mm	3: Pullulan + BMP-2 (1 µg) (N=NS)			
		H: NS	4: Pullulan + BMP-2 (1.5 µg) (N=NS)			
			4: Pullulan + BMP-2 (2 µg) (N=NS)		HE staining; Von Kossa staining (qualitative analysis)	Pullulan + BMP-2 stimulation of osteoblast activity to form new bone
	Mouse (male, ICR), *n* = NS	Calvaria	1: Empty (N=NS)	4 W	Micro-CT (qualitative analysis)	Pullulan alone failed to repair bone defect
		Ø: 4.6 mm	2: Pullulan (N=NS)			Pullulan + BMP-2 (2 µg) full repair of bone defect
		H: NS	3: Pullulan + BMP-2 (1 µg) (N=NS)			
			4: Pullulan + BMP-2 (2 µg) (N=NS)		HE staining (qualitative analysis)	New bone formation at the edges of the defect for Pullulan + BMP-2 groups
Miyahara et al. (2011)	Rat (NS, Wistar), *n* = 36	Calvaria	1: Empty (N = 24)	2 W	Micro-CT (BV in mm^3^)	At 4W: Pullulan membrane * > Collagen membrane
		Ø: 5 mm	2: Collagen membrane (N = 24)	4 W	HE staining (qualitative analysis)	Collagen membrane: immature bone synthesized on both sides of the membrane
		H: NS	3: Pullulan membrane (N = 24)	8 W		Pullulan membrane: Mature bone synthesized; regeneration occurred under the membrane
Fujioka-Kobayashi et al. (2012)	Mouse (NS, C57BL/6N), *n* = 43	Calvaria	1: Pullulan + PBS (N = 11)	0 W	Micro-CT (Bone healing in %)	At 8W: Pullulan + FGF18 + BMP-2 * > Pullulan + BMP-2
		Ø: 3 mm	2: Pullulan + FGF18 (N = 10)	1 W		
		H: 5 mm	3: Pullulan + BMP-2 (N = 11)	2 W		
			4: Pullulan + FGF18 + BMP-2 (N = 11)	3 W	Alizarin Red and Calcein staining (qualitative analysis)	Newly bone synthesis in the defect in Pullulan + BMP-2 and Pullulan + FGF18 + BMP-2 groups
				6 W		
				8 W		
	Mouse (NS, Osx1-GFP::Cre/B26B), *n* = 6	Calvaria	1: Pullulan-Rh + PBS (N = 3)	2 W	X-gal staining	Osterix 1 gene activated for the formation of new bone in Pullulan-Rh + FGF18 + BMP-2 group
		Ø: 3 mm	2: Pullulan-Rh + FGF18 + BMP-2 (N = 3)			
		H: NS				
Takahata et al. (2015)	Rabbit (female, New Zealand), *n* = 6	Ulnar (diaphysis)	1: α-TCP (N=NS)	4W	HE staining; Safranin O staining (qualitative analysis)	Mature bone formation for Pullulan + β-TCP group
		L: 10 mm	2: Pullulan + β-TCP (N=NS)	8W		Fragmented bone ingrowth for α-TCP group
	Mouse (female, C57BL/6J), *n* = 13	Femoral (Intramedular injection) Size: NA	1: Empty (N = 4)	4W	Micro-CT (BMD in mg/cm^3^)	Pullulan + β-TCP * > Empty
			2: Pullulan (N = 4)			
			3: Pullulan + β-TCP (N = 4)			
			1: Empty (N = 3)	2 W	HE staining (qualitative analysis)	At 5W: bone formation began for Pullulan + β-TCP group
			2: Pullulan (N = 3)	5 W		
			3: Pullulan + β-TCP (N = 3)	8 W		
	Pig (female, NS), *n* = 4	Vertebral body Size: 1 cm^2^	1: Empty (N=NS)	8 W	Micro-CT (CT number in Hounsfield Unit)	Pullulan + β-TCP ** > Empty
			2: α-TCP (N=NS)			
			3: Pullulan + β-TCP (N=NS)			
			1: α-TCP (N=NS)	4 W	HE staining (qualitative analysis)	At 4W: bone defect recovered by bone healing for Pullulan + β-TCP group whereas α-TCP group demonstrated fragmented bone ingrowth
			2: Pullulan + β-TCP (N=NS)	8 W		
Charoenlarp et al. (2018)	Mouse (male, C57BL/6N), *n* = 6–10 per condition	Calvaria	1: Pullulan (air) + PBS (N=NS)	0 W	Micro-CT (% of healing)	At 8W: Pullulan (cross-linked RGD) + BMP-2 + FGF18 * > Pullulan (freeze-dried) + BMP-2 + FGF18
		Ø: 3 mm	2: Pullulan (freeze-dried) + PBS (N=NS)	1 W		
		H: NS	3: Pullulan (cross-linked RGD) + PBS (N=NS)	2 W		
			4: Pullulan (air) + BMP-2 + FGF18 (N=NS)	3 W		
			5: Pullulan (freeze-dried) + BMP-2 + FGF18 (N=NS)	4 W		
			6: Pullulan (cross-linked RGD) + BMP-2 + FGF18 (N=NS)	6 W		
				8 W	Modified Tetrachrome staining (qualitative analysis)	Whether Pullulan was cross-linked with RGD peptides or freeze-dried and combined with BMP-2 and FGF18, it showed trabecular bone formation with calcified bone fragmented
	Mouse (male, ICR), *n* = 3–4 per condition	Calvaria	1: Pullulan (cross-linked RGD) + PBS (N=NS)	1 W	Modified Tetrachrome staining (qualitative analysis)	Presence of osteoblasts in Pullulan (cross-linked RGD) + BMP-2 + FGF18 and bone regeneration begun under the nanogel
		Ø: 3 mm	2: Pullulan (cross-linked RGD) + BMP-2 + FGF18 (N=NS)			
		H: NS				
Popescu et al. (2019)	Rat (male, Wistar), *n* = 30	Femoral (diaphysis, unicortical)	1: Alginate/Pullulan (N=NS)	0 D	MRI (Bone defect width in µm)	All groups showed a decrease in bone defect size but it was faster for Alginate/Pullulan/1.5CuBG group (No statistical comparison done)
			2: Alginate/Pullulan + β-TCP/HA (N=NS)	5 D		
			3: Alginate/Pullulan/0.5CuBG (N=NS)	28 D		
		W: 2 mm	4: Alginate/Pullulan/1.5CuBG (N=NS)	0 D	HE staining (qualitative analysis)	Alginate/Pullulan/0.5 or 1.5CuBG showed osteoid tissue formation with parallel organized fibers
			5: Alginate/Pullulan/BG (N=NS)	35 D		

BG, bioglass; BMD, bone mineral density; BMP, bone morphogenic protein; BV, bone volume; β-TCP, beta-tricalcium phosphate; H, height; HA, hydroxyapatite; HE, hematoxylin and eosin; L, length; Micro-CT, micro-computed tomography; NA, not applicable; NaCl sodium chloride; NS, not specified; PBS, phosphate buffer saline; Rh rhodamine; W, width; X-gal 5-bromo-4-chloro-3-indolyl-b-D-galactopyranoside; * *p* < 0.05; ** *p* < 0.01; *** *p* < 0.001; ø, diameter.

**TABLE 5:** Preclinical studies using Dextran and Pullulan scaffolds for bone regeneration in orthotopic sites.

Author (Year)	Animal model (sex; species); n, number of animals used	Type of defect (defect size)	Conditions (N, number of defects created in total per condition)	Observation time [in day (D), week (W) or month (M)]	Experimental analysis	Results
Fricain et al. (2013)	Rat (NS, Wistar), *n* = NS	Femoral (condyle)	1: Empty (N = 6)	15 D	Micro-CT (MC in mg; MD in mg/cm^3^)	Pullulan/Dextran + HA * > Pullulan/Dextran * > Empty
		Ø: 5 mm	2: Pullulan/Dextran (N = 18)	30 D		
		H: 6 mm	3: Pullulan/Dextran + HA (N = 18)	90 D	Von Kossa staining; Masson’s Trichrome staining (qualitative analysis)	Tissue mineralization was more important for Pullulan/Dextran + HA than Pullulan/Dextran
	Goat (NS), *n* = 7	Mandibular	1: Empty (N = 2)	1 M	Micro-CT (qualitative analysis)	Osteoid tissue formation observed for Pullulan/Dextran + HA group
		Ø: 10 mm	2: Pullulan/Dextran + HA (N = 10)	6 M		
		H: 8 mm			Von Kossa staining; Masson’s Trichrome staining (qualitative analysis)	New osteoid tissue formation for Pullulan/Dextran + HA group and mineralized tissue
		Tibial (epiphysis)	1: Empty (N = 2)	1 M	Micro-CT (qualitative analysis)	Mineralized tissue within the defect for Pullulan/Dextran + HA group
		L: 40 mm	2: Pullulan/Dextran + HA (N = 10)	6 M		
		W: 12 mm			Von Kossa staining; Masson’s Trichrome staining (qualitative analysis)	Induction of mineralized tissue with organized lamellar bone by Pullulan/Dextran + HA group
Schlaubitz et al. (2014)	Rat (female, Wistar RjHan), *n* = 18	Femoral (condyle)	1: Empty (N = 18)	15 D	Micro-CT (BMC in mg; BMD in mg/cc)	Pullulan/Dextran + HA ** > Empty
		Size: 38 mm^3^	2: Pullulan/Dextran + HA (N = 18)	30 D	Von Kossa staining (osteoid within the region of interest in %)	Pullulan/Dextran + HA ** > Empty
				70 D		
Frasca et al. (2017)	Rat (male, Lewis), *n* = 90	Femoral metaphysis	1; Empty (N = 30)	7 D	Micro-CT (BV/TV in %)	At 30D: Pullulan/Dextran + MSCs* > MSCs
		Ø: 3 mm	2: MSCs (N = 30)	30 D		
		H: 5 mm	3: Pullulan/Dextran (N = 30)	90 D		
			4: Pullulan/Dextran + MSCs (N = 30)			
			5: HA/β-TCP (N = 30)		Von Kossa staining; Masson’s Trichrome staining (qualitative analysis)	For Pullulan/Dextran with or without MSCs groups, formation of trabecular and cortical bones
			6: HA/β-TCP + MSCs (N = 30)			
Ribot et al. (2017)	Rat (female, Wistar RjHan), *n* = 33	Femoral condyle	1: Pullulan/Dextran + Fucoidan (N = 15)	1 W	MRI (Volume of hyper intense signal in mm^3^)	At 3W: Pullulan/Dextran + Fucoidan > Pullulan/Dextran + Fucoidan + HA * > Pullulan/Dextran + HA *
			2: Pullulan/Dextran + HA (N = 12)			
			3: Pullulan/Dextran + Fucoidan + HA (N = 18)			
		Ø: 3,5 mm	1: Pullulan/Dextran + Fucoidan (N = 24)	3 W		
		H: 4 mm	2: Pullulan/Dextran + HA (N = 24)	5 W	Micro-CT (BV/TV in %)	At 5W: Pullulan/Dextran + HA** > Pullulan/Dextran + Fucoidan + HA** > Pullulan/Dextran + Fucoidan
			3: Pullulan/Dextran + Fucoidan + HA (N = 30)		Masson’s Trichrome staining (% of mature bone per defect)	At 3W: Pullulan/Dextran + HA * > Pullulan/Dextran + Fucoidan + HA * > Pullulan/Dextran + Fucoidan
Fricain et al. (2018)	Sheep (NS), *n* = 12	Maxillary sinus (i.e. sinus lift procedure)	1: Bio-Oss® (N = 12)	0 M	Micro-CT (MV/TV ratio)	Pullulan/Dextran + HA ≈ Bio-Oss® (n/s)
			2: Pullulan/Dextran + HA (N = 12)	3 M		
				6 M	Masson’s Trichrome staining (Bone tissue in mm^2^)	Pullulan/Dextran + HA ≈ Bio-Oss® *
Maurel et al. (2021)	Rat (female, NS), *n* = 6	Femoral condyle	1: Pullulan/Dextran + HA resuspended in NaCl 0.9% (N = 6)	30 D	Micro-CT (BV/TV ratio)	No significant difference between the two groups
		Ø: 4 mm	2: Pullulan/Dextran + HA resuspended in autologous blood (N = 6)	60 D	Masson’s Trichrome staining (new bone surface in %)	Osteoid tissue formation with trabecular-like structures for both conditions
		H: 6 mm				
	Sheep (NS), *n* = 3	Maxillary sinus (i.e. sinus lift procedure)	1: Pullulan/Dextran + HA resuspended in NaCl 0.9% (N = 3)	3 M	Cone Beam Computer Tomography (MV/TV ratio)	No significant difference between the two groups
			2: Pullulan/Dextran + HA resuspended in autologous blood (N = 3)		Masson’s Trichrome staining (new bone surface in %)	Osteoid tissue formation for both conditions

BMC, bone mineral content; BMD, bone mineral density; BV/TV, bone volume over total volume; β-TCP, beta-tricalcium phosphate; H, height; HA, hydroxyapatite; L, length; MC, mineral content; MD, mineral density; Micro-CT, micro-computed tomography; MRI, magnetic resonance imaging; MSCs, mesenchymal stromal cells; MV/TV, mineral volume over total volume; NS, not specified; NaCl, sodium chloride; * *p* < 0.05; ** *p* < 0.01; ø, diameter.

### 3.3 Polysaccharide-Based Materials

Among the 28 studies included, 24 used either dextran or pullulan as the main component for biomaterial design (Lafont et al., 2004; Chen et al., 2005; Maire et al., 2005a; Chen et al., 2006; Chen et al., 2007; Degat et al., 2009; Hayashi et al., 2009; Abbah et al., 2012; Fujioka-Kobayashi et al., 2012; Miyahara et al., 2012; Bölgen et al., 2014; Takahata et al., 2015; Charoenlarp et al., 2018; Ritz et al., 2018; Chen et al., 2019; Ding et al., 2019; Fang et al., 2019; Popescu et al., 2020) and six used a combination of these two polysaccharides (Fricain et al., 2013; Schlaubitz et al., 2014; Frasca et al., 2017; Ribot et al., 2017; Fricain et al., 2018) (Table 1). In most cases, when pullulan or dextran was used alone, chemical functionalization of the polysaccharide was performed to improve the cross-linking process and/or to promote their binding capacity to growth factors. For dextran derivatives, acrylate groups (Chen et al., 2006; Chen et al., 2007) (e.g., glycidyl methacrylate, urethane methacrylate), amine or amide derivatives (Lafont et al., 2004; Maire et al., 2005a; Degat et al., 2009; Abbah et al., 2012; Shoji et al., 2020) (e.g., benzylamide, diethylaminoethyl, tyramine), carboxyl (Lafont et al., 2004; Degat et al., 2009; Ritz et al., 2018) (e.g., carboxylate, carboxymethyl, formylbenzoic), aldehyde (Wang et al., 2021), catechol (Wang et al., 2021) (i.e., dopamine), or sulfated groups (Chen et al., 2019) were added to the dextran backbone in 11 studies. Concerning pullulan derivatives, acrylate groups (Hayashi et al., 2009; Fujioka-Kobayashi et al., 2012; Miyahara et al., 2012; Charoenlarp et al., 2018) (e.g., acryloyl, cholesteryl) or phosphate groups (Takahata et al., 2015) were added for functionalization in five studies. No chemical modification was reported for compositions using both polysaccharides (Fricain et al., 2013; Schlaubitz et al., 2014; Frasca et al., 2017; Ribot et al., 2017; Fricain et al., 2018; Maurel et al., 2021).

Physical cross-linking and chemical cross-linking were used for hydrogels (Kumari and Badwaik, 2019). Physically cross-linked of dextran-derived biomaterials were identified in two studies by using ionic cross-linking [e.g., calcium chloride (Abbah et al., 2012)] or by micellar reaction [e.g., sodium dodecyl sulfate/stearyl methacrylate (Fang et al., 2019) (SDS/SMA) micelles]. Chemical cross-linking was the most commonly used procedure. For chemically cross-linked dextran and/or pullulan-derived biomaterials, a wide range of reagents and reactions were employed, such as methylenebisacrylamide (Bölgen et al., 2014) (MBAm), polyethylene glycol (Chen et al., 2006) (PEG), thiol group-modified polyethylene glycol (Hayashi et al., 2009; Fujioka-Kobayashi et al., 2012; Miyahara et al., 2012; Charoenlarp et al., 2018) (PEGSH), PEGSH with “arginine-glycine-aspartate” (RGD) peptides (Charoenlarp et al., 2018), sodium trimetaphosphate (Maire et al., 2005a; Fricain et al., 2013; Schlaubitz et al., 2014; Frasca et al., 2017; Ribot et al., 2017; Fricain et al., 2018; Maurel et al., 2021) (STMP), through Schiff reaction (imine bonding) (Wang et al., 2021), through enzymatic reaction (Shoji et al., 2020) (e.g., hydrogen peroxide, horseradish peroxidase) or by activating a photocross-linking group using UV light (Ritz et al., 2018).

We have identified three main routes of administration of these biomaterials in implantation sites. They could be implanted as an injectable hydrogel or microspheres/microbeads (e.g., dextran and/or pullulan-derived biomaterials), as an aqueous solution adsorbed on a collagen or a polyvinyl alcohol sponge [e.g., dextran-derived biomaterials (Lafont et al., 2004; Degat et al., 2009; Togami et al., 2015; Chen et al., 2019; Yu et al., 2020)] or as a membrane for guided bone regeneration [e.g., pullulan-derived biomaterial (Miyahara et al., 2012)].

**FIGURE 5:**
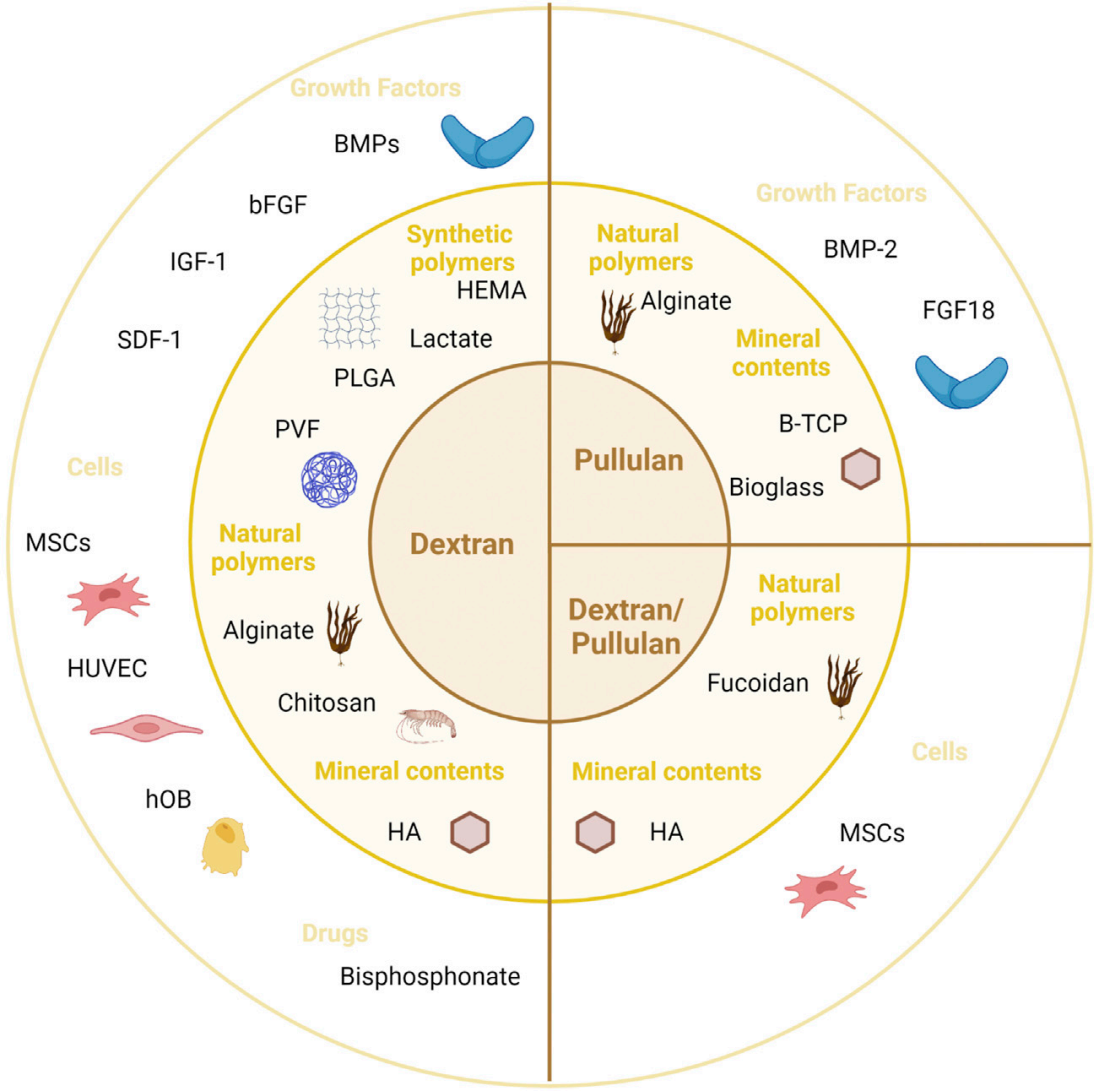
Dextran and/or pullulan-based scaffolds for bone tissue engineering applications. Created with BioRender.com. bFGF, basic fibroblast growth factor; BMP, bone morphogenetic protein; B-TCP, beta-tricalcium phosphate; FGF, fibroblast growth factor; HA, hydroxyapatite; HEMA, hydroxyethyl methacrylate; hOB, human osteoblast; HRP, horseradish peroxidase; HUVEC, human umbilical vein endothelial cells; IGF, insulin-like growth factor; MSCs, mesenchymal stromal cells; PLGA, poly-(L-glutamic acid); PVF, polyvinyl formal; SDF, stromal-derived growth factor.

To induce or enhance bone formation, various elements were directly added in these scaffolds to generate composite scaffolds (Figure 5). Bioceramics, such as hydroxyapatite (HA) (Fricain et al., 2013; Schlaubitz et al., 2014; Ribot et al., 2017; Fricain et al., 2018; Ding et al., 2019; Fang et al., 2019; Maurel et al., 2021; Wang et al., 2021), β-tricalcium phosphate (Takahata et al., 2015) (β-TCP) or bioactive glass (Popescu et al., 2020) were thus added to the scaffolds in nine studies, whereas another natural or synthetic polymer was combined to the scaffold in nine studies (Chen et al., 2005; Chen et al., 2006; Chen et al., 2007; Abbah et al., 2012; Togami et al., 2015; Ding et al., 2019; Fang et al., 2019; Popescu et al., 2020; Wang et al., 2021).

Another reported strategy to enhance bone formation was the incorporation of bioactive molecules to these biomaterials to create a delivery system. Growth factors were employed in 13 studies, namely, bone morphogenetic proteins (Chen et al., 2005; Maire et al., 2005a; Chen et al., 2007; Degat et al., 2009; Hayashi et al., 2009; Abbah et al., 2012; Fujioka-Kobayashi et al., 2012; Charoenlarp et al., 2018; Chen et al., 2019; Yu et al., 2020) (e.g., BMP-2 and BMP-7), basic fibroblast growth factor (Shoji et al., 2020) (bFGF), fibroblast growth factor (Fujioka-Kobayashi et al., 2012; Charoenlarp et al., 2018) (FGF18), insulin-like growth factor (Chen et al., 2006) (IGF-1) and stromal-derived growth factor (Ritz et al., 2018) (SDF-1). One study reported the incorporation into the scaffold of bisphosphonate, which exhibits strong affinity with hydroxyapatite (Wang et al., 2021).

Incorporation of cells into the polysaccharide scaffolds was the last identified procedure to promote bone regeneration (three studies). Three types of cells have been incorporated to scaffolds to promote bone regeneration: rat mesenchymal stromal cells (Bölgen et al., 2014; Frasca et al., 2017) (MSCs), human umbilical vein endothelial cells (Ritz et al., 2018) (HUVEC), or human osteoblasts (Ritz et al., 2018).

For all these different strategies, the performance for regenerating bone tissue is examined in the following sections.

### 3.4 Evaluation of Performance of Polysaccharide-Based Materials for Bone Formation

#### 3.4.1 Dextran-Based Materials for Bone Regeneration

Twelve studies investigated orthotopic bone formation by using dextran-based biomaterials and four studies investigated ectopic bone formation in rodents (Tables 2, 3).

##### 3.4.1.1 Dextran-Based Materials as a Bone Graft Substitute

To determine bone regeneration ability of dextran-derived biomaterials, several studies combined this polysaccharide with natural or synthetic polymers. For example, polyvinyl formal (PVF) sponges have been coated with dextran to enhance the poor osteogenic ability of this synthetic polymer due to its fibrous construction (Togami et al., 2015). Investigation of the water holding capability of the scaffold revealed that dextran-coated PVF sponge with high water capability significantly improved bone formation in a rat femoral defect.

Three studies assessed the osteogenic capability improvement of dextran-based hydrogels loaded with mineral particles. Dextran-based hydrogel combined with a synthetic polymer (polyacrylamide) loaded or not with hydroxyapatite (HA) particles was implanted into a rabbit femoral defect (Fang et al., 2019). Bone regeneration was significantly improved in the presence of HA. In another case, dextran was mixed to chitosan with strontium-doped mineral particles to further improve bone regeneration (Ding et al., 2019). Nanohydroxyapatite particles doped with increasing molar ratios of strontium (0%, 50%, and 100%) were incorporated into the polymeric mixture and implanted in a rat calvarial defect model. The adjunction of nanohydroxyapatite doped with 100% strontium significantly enhanced bone regeneration, whereas the dextran-based hydrogel without strontium-nanohydroxyapatite showed the lowest regeneration rate.

Finally, an injectable hydrogel composed of dextran and poly (L-glutamic acid) inspired from mussel adhesion to design an adhesive, self-healing biomaterial with osteogenic properties through HA and bisphosphonate incorporation inside the scaffold was designed by Wang et al. (2021). Scaffolds were implanted in a rat calvarial defect and showed a significant enhancement of bone regeneration compared with control.

##### 3.4.1.2 Dextran-Based Material to Create Heparan Sulfate Mimetic Molecules (i.e., Regenerating Agent, RGTA®)

Regenerating Agents (RGTA®) are polysaccharides designed to replace altered heparan sulfate in injured tissues (Barritault et al., 2017). They are derived from dextran that is chemically modified by sulfate and carboxyl groups. One of these RGTA®, derived from dextran, was investigated for bone regeneration in a rat calvarial defect model (Lafont et al., 2004). Where dextran backbone was functionalized with methylcarboxyl, benzylamide, and sulfate groups. Two conditions were applied on a collagen sponge either with a solution of dextran derivatives like RGTA® or with PBS. Bone healing occurred earlier with the dextran derivative at 7 days after implantation.

##### 3.4.1.3 Dextran-Based Material as a Potential Cell Carrier System

Scaffold designed for BTE loaded with cells can act as a carrier system if cells loaded on the scaffold stay inside to recruit endogenous cells, or can act as a cell delivery system if cells migrate to the surrounding tissue (Lalande et al., 2011). Two studies reported the use of dextran-derived biomaterials as a carrier for cell delivery (Bölgen et al., 2014; Ritz et al., 2018). A dextran-based cryogel was implanted in a rat calvarial defect loaded with or without stem cells (rat MSCs) (Bölgen et al., 2014). Bone regeneration significantly increased over time in both conditions. However, no significant difference was evidenced between the “scaffold-stem cells” group and the cell-free scaffold group whatever the time point (30 and 90 days). These results were consistent with another study focusing on a dextran-based material loaded with HUVECs and osteoblasts*.* (Ritz et al., 2018). Dextran-based hydrogels were either loaded with a monoculture of HUVECs or a co-culture of human osteoblasts with HUVECs. Results at 8 weeks showed that hydrogels loaded with HUVECs significantly enhanced bone formation compared with the defect left empty. However, this study showed no significant difference between the “scaffold-cells” group and the cell-free scaffold group, thereby demonstrating that a use of dextran-based hydrogels as a cell carrier system to deliver rat MSCs or HUVECs fail to enhance dextran osteogenic properties.

##### 3.4.1.4 Dextran-Based Material as a Growth Factor Carrier

Dextran-derived biomaterials were also reported as growth factor carriers to promote bone regeneration. As previously mentioned, dextran polysaccharide chains have been chemically modified by amine, amide, carboxyl or acrylic groups to promote growth factor delivery system. Those modifications were performed either to 1) cross-link the polymeric scaffold to enable the encapsulation of growth factors or 2) to directly interact with them.

Four studies investigated the osteoinductive potential of dextran-based materials as a delivery growth factor system in ectopic bone formation models (Maire et al., 2005a; Degat et al., 2009; Chen et al., 2019; Yu et al., 2020) (Table 2).

Maire et al. (2005a) used modified dextran with different degrees of substitution of hydroxyl groups (i.e., number of modified groups adding per 100 glucose units) and combined extracted bovine BMP-2 (0.5 and 5 µg) to analyze ectopic bone formation and calcification. Modification of the dextran backbone enabled cross-linking of the scaffold. It appeared that depending on the functionalization rate of dextran (e.g., degree of substitution of 0%, 2% or 18% w/w), BMP-2 was gradually delivered to the surrounding environment to promote bone formation and calcification. The highest growth factor retention was observed using the highest functionalized dextran content (D.S. = 18%). Small concentration of BMP (0.5 µg) failed to induce bone formation regardless of the functionalization rate.

In another study, the number of carboxymethyl and benzylamide groups (i.e., for cross-linking purpose) on dextran backbone was modified to investigate subcutaneously BMP-2 delivery (Degat et al., 2009). A dose-dependent trend was observed: 1 or 10 µg of modified dextran were sufficient to induce ectopic bone formation whereas a higher concentration of this dextran derivative (100 µg) inhibited such formation.

The impact of sulfated groups on different polymers (e.g., chitosan, dextran) and its ability to release rhBMP-2 (10 µg) was determined in a mouse muscle implantation model (Chen et al., 2019). Dextran substitutes improved binding with cytokine (e.g., rhBMP-2) through electrostatic interactions but generated few bones ectopically compared with chitosan. It was hypothesized that the modified dextran having less sulfated groups than the modified sulfated chitosan, it failed to properly deliver rhBMP-2 and induce bone formation.

The last study in ectopic model by Yu et al. (2020) *.* confirmed these findings by loading unmodified dextran with rhBMP-2. It appeared that dextran scaffold generated few trabecular structures after 8 weeks.

Five studies reported the use of dextran-based materials as a delivery growth factor in orthotopic bone models. Dextran-based microbeads modified with an amino group, diethylaminoethyl (DEAE) was investigated in a rat arthrodesis model (Abbah et al., 2012). Dextran-based scaffold failed to successfully deliver BMP-2 (5 µg) and to induce bone formation as shown by micro-CT analysis. In comparison, another amino group (i.e., tyramine) was used to modify dextran backbone in a mice fracture model (Shoji et al., 2020). This association has improved the delivery of b-FGF and accelerate bone formation. Large calluses and new bone areas were observed at the fracture site.

Another team assessed the potential of dextran/gelatin scaffolds to act as a delivery growth factors system [e.g., BMP-2 (Chen et al., 2005; Chen et al., 2007) or IGF-1 (Chen et al., 2006)] in a periodontal defect model on dogs. Chemical properties of dextran/gelatin scaffold were investigated by functionalized dextran (Chen et al., 2006), and growth factors entrapment inside the scaffold (Chen et al., 2005; Chen et al., 2007). Here, the functionalization of dextran backbone only played a role on the reticulation process of the biomaterial. In their first study (Chen et al., 2005), dextran/gelatin microspheres were combined with BMP-2 and mixed in CPC to fill periodontal defect. In addition, a chitosan membrane loaded with these microspheres was added to cover the defect. Compared with the control group (i.e., CPC covered by chitosan membrane), dextran/gelatin microspheres covered with the chitosan membrane presented new bone formation 8 weeks after surgery.

Another approach concerns the dextran functionalization with glycidyl methacrylate at different degrees of substitution (e.g., 4.7, 6.3, and 7.8) combined with IGF-1 (Chen et al., 2006). Dextran with the smallest rate of substitution (D.S. = 4.7) showed positive results to properly deliver IGF-1 and then regenerate bone.

Chen et al. (2007) investigated whether BMP-2 had to be adsorbed or loaded through microsphere encapsulation in the dextran/gelatin scaffold. BMP-2 activity seemed to be better preserved when loaded in dextran/gelatin microspheres in the scaffold compared when it was adsorbed at the surface of the material.

Finally, dextran scaffold was successfully loaded with stromal-derived growth factor (SDF-1) in a mice calvarial defect. Bone regeneration was significantly improved compared with the empty defect (Ritz et al., 2018), but without significant difference with the growth factor-free scaffold.

#### 3.4.2 Bone Formation With Pullulan Substitutes

Six studies investigated orthotopic bone formation by using pullulan-based biomaterials and one study assessed ectopic bone formation in rodents (Tables 2, 4).

##### 3.4.2.1 Pullulan-Based Materials as a Bone Graft Substitute

Numerous studies described the performance of composite materials with pullulan. Combination of pullulan to alginate and bioactive glasses with various percentage of copper oxide to regenerate bone was investigated in a rat femoral defect and was compared with a commercial β-TCP/HA substitute embedded in an alginate-pullulan composite scaffold (Popescu et al., 2020). A progressive healing of bone was observed whatever the tested scaffold composition. Interestingly, a regeneration process was also observed when the alginate–pullulan composite materials were implanted in such orthotopic site without an osteoconductive component (i.e., β-TCP/HA and bioglass), thereby suggesting the potential of these materials for BTE applications. However, there was no empty group as a control in this study.

Phosphorylated-pullulan mixed with β-TCP had been tested in three different models, a rabbit ulnar defect, a pig vertebral defect, and a mouse femoral injection (Takahata et al., 2015). It showed similar bone regeneration compared to a clinical bone substitute made of α-TCP (Biopex-R®) in a rabbit ulnar bone defect model. Additionally, implantation of the composite scaffold induced new bone formation at 4 and 8 weeks, whereas Biopex-R® remained isolated from the surrounding bone at 8 weeks.

##### 3.4.2.2 Pullulan-Based Material as a Potential Growth Factor Carrier

Three studies mentioned the substitution of pullulan main chains by cholesteryl and acryloyl groups to proceed cross-linking between pullulan macromolecules and then to establish a growth factor delivery system (Hayashi et al., 2009; Fujioka-Kobayashi et al., 2012; Charoenlarp et al., 2018). Modified pullulan successfully delivered BMP-2 (2 µg) and induced bone formation ectopically in a mice model (Hayashi et al., 2009). The potential of this scaffold to deliver BMP-2 and promote bone regeneration by varying BMP-2 concentration was then determined in an orthotopic mouse calvarial model. Implantation of pullulan-based hydrogel containing 2 µg of BMP-2 showed the best results which was evidenced by the complete healing of the critical defect. One study described the effect of a co-administration of FGF18 and BMP-2 implanted in a bone defect using a pullulan-based hydrogel (Fujioka-Kobayashi et al., 2012). The amount of newly formed bone was higher when both growth factors were delivered, suggesting that this system improved the efficiency of BMP2-dependent bone healing in a mouse calvarial defect model.

Charoenlarp et al. (2018) also used pullulan-based materials to act as a delivery system for multiple growth factors (i.e., BMP-2 and FGF18). They assessed their growth factor release pattern and observed an initial burst followed by a gradual sustained release more than 1 week. They also observed that functionalization with RGD peptides during synthesis of gels enhanced bone healing as growth factors interact with the repeated units.

##### 3.4.2.3 Pullulan-Based Membrane for Guided Bone Regeneration

A nanogel membrane made with cholesteryl and acryloyl-binding pullulan was evaluated for guiding bone regeneration (Miyahara et al., 2012). This nanogel membrane was compared with a commercially available collagen membrane (Koken Tissue Guide®) in a rat calvarial defect and they both stimulated bone regeneration compared with the control, in which no membrane was applied. Earlier bone regeneration was significantly enhanced with the pullulan-based nanogel membrane and newly formed bone was more mature 2 weeks after surgery.

#### 3.4.3 Pullulan/Dextran-Based Materials for Bone Regeneration

Six studies investigated orthotopic bone formation by using pullulan/dextran-based biomaterials in which one study also assessed ectopic bone formation in rodents and goats (Tables 2, 5).

##### 3.4.3.1 Pullulan/Dextran-Based Substitute Used as a Bone Graft Substitute

One team focused on a composite scaffold combining a pullulan/dextran-based material with HA particles to promote osteogenesis (Fricain et al., 2013; Schlaubitz et al., 2014; Ribot et al., 2017; Fricain et al., 2018; Maurel et al., 2021). Firstly, this biomaterial was supplemented or not with hydroxyapatite in heterotopic and orthotopic sites on mice and goat to investigate the osteoinductive and osteoconductive properties (Fricain et al., 2013). Subcutaneous and intra-muscular implantations on small and large mammals revealed that this pullulan/dextran scaffold combined with HA enabled osteoid tissue formation. Besides, the composite scaffold induced a highly mineralized tissue in three different bony sites (e.g., femur, mandible, and tibia), as well as osteoid tissue and bone tissue regeneration in direct contact to the matrix. The same composite scaffold was then designed as cross-linked microbeads to be implanted in a rat femoral defect (Schlaubitz et al., 2014). Bone regeneration was significantly enhanced compared with the empty group. Interestingly, one study compared in a maxillary bone defect in sheep this pullulan/dextran-based scaffold with HA to a widely used clinical xenograft (BioOss®) (Fricain et al., 2018). Similar results were obtained for both materials. These composite microbeads were also either reconstituted in saline buffer or autologous blood to investigate the role of this vehicle for bone regeneration (Maurel et al., 2021). They displayed important mineralization process without significant difference, thereby suggesting that reconstitution of microbeads with autologous blood is not required. A study focused on the interest of Magnetic Resonance Imaging (MRI) for longitudinal evaluation of three different biomaterials based on pullulan/dextran and containing either fucoidan and/or HA for bone regeneration in a rat femoral bone defect (Ribot et al., 2017). The high sensitivity of MRI showed that the material without HA was the least efficient for bone regeneration, which was confirmed by micro-CT images and histology. After 5 weeks, pullulan/dextran-based scaffold containing either HA alone or Fucoidan plus HA showed similar results.

##### 3.4.3.2 Pullulan/Dextran-Based Material as a Potential Stem Cell Vehicle

Pullulan/dextran polysaccharide-based scaffold supplemented with MSCs was compared with a commercially available CPC bone substitute (Calciresorb C35®) (Frasca et al., 2017) for their ability to promote bone regeneration once loaded with cells. They were implanted alone or combined with syngenic MSCs from rat bone marrow. After 1 month, MSCs combined with these biomaterials significantly enhanced bone healing compared with their respective group without MSCs. After 3 months, bone regeneration was significantly enhanced for each condition without difference between cellularized and non-cellularized biomaterials. Results also showed that pullulan/dextran substitutes had a better resorption rate than CPC particles.

## 4 Discussion

The purpose of this work was to review the design of pullulan and/or dextran-derived biomaterials used for bone regeneration. Polysaccharide scaffolds were systematically analyzed. These scaffolds were mostly used as a bone substitute (13 studies) or as a growth factor delivery system (13 studies). Three studies investigated the ability of these polysaccharide-based scaffolds to act as cell carriers for BTE applications and only one study suggested the use of a pullulan-based nanogel as a membrane for guided bone regeneration (Miyahara et al., 2012). The present systematic review showed that most of the pullulan-based and dextran-based materials underwent functionalization methods for BTE applications. Chemical functionalization is the most widely used approach to improve reticulation process and/or to promote their binding capacity to growth factors, thus enhancing their potential to act as growth factors carrier. The introduction of charged groups also seemed to provide binding sites for host cells to adhere to the material (Filippi et al., 2020). Addition of acrylate groups (e.g., acryloyl, cholesteryl) was the most reported process to functionalize pullulan-based hydrogel (Hayashi et al., 2009; Fujioka-Kobayashi et al., 2012; Miyahara et al., 2012; Charoenlarp et al., 2018). Hydrogels of cholesterol-bearing pullulan are already considered as unique materials for various drug delivery applications (Morimoto et al., 2005a; Kato et al., 2007). Combination of cholesteryl group-associated hydrophobic domains and hydrophilic polysaccharide chains provides an amphiphilic hydrogel that shows effective drug-trapping sites in itself (Akiyoshi et al., 1998).

A wide range of functionalization methods were used for dextran-based materials, making it impossible to select a specific one for these polysaccharide-based materials. Interestingly, no chemical modification of pullulan/dextran-based matrix was also reported (Fricain et al., 2013; Schlaubitz et al., 2014; Frasca et al., 2017; Ribot et al., 2017; Fricain et al., 2018; Maurel et al., 2021).

This review also highlights the significant use of cross-linking reagents for these polysaccharide-based biomaterials synthesis. Cross-linkers have attracted much attention to enhance the biological functionality and mechanical properties of biopolymers (Oryan et al., 2018; Krishnakumar et al., 2019). Cross-linking strategies vary depending on the chemical nature of the biomaterials. This review emphasized that chemical cross-linkers were extensively used in the included studies. This is consistent with previous studies that establish that chemical cross-linking is the most commonly employed strategy to develop bone substitutes (Krishnakumar et al., 2019). For instance, successful chemical cross-linking of pullulan/dextran-based materials was carried out using the cross-linking agent STMP (Fricain et al., 2013; Schlaubitz et al., 2014; Frasca et al., 2017; Ribot et al., 2017; Fricain et al., 2018; Maurel et al., 2021). STMP is a nontoxic cross-linker, already used in the food industry (to cross-link starch) or for pharmaceutical applications (for hydrogels synthesis) (Gliko-Kabir et al., 2000; Lack et al., 2007). The present review outlined that pullulan/dextran-based materials were cross-linked with STMP. However, it should be mentioned that all the works carried out on pullulan/dextran-based materials comes from the same group. Pullulan-based materials were successfully cross-linked with thiol-bearing in four studies (Hayashi et al., 2009; Fujioka-Kobayashi et al., 2012; Miyahara et al., 2012; Charoenlarp et al., 2018), whereas no cross-linking reagents were used in two studies. Finally, cross-linking strategies were less employed to functionally modify dextran-based materials (only half of the included studies). Furthermore, each study focused on a different cross-linking process, making it difficult to draw conclusions.

Biomaterial design also plays a key role in promoting bone healing. In the present review, we outlined that these polysaccharide-derived biomaterials are mainly used as hydrogels or microbeads. A growing interest for polymer hydrogels in BTE is arising (Tang et al., 2021) as they exhibit several promising properties in a context of bone repair: they are ready-to-use material that can be molded to any shape, size, or form, thereby fitting easily in the bone defect (Li et al., 2021). Six studies designed disc-shaped hydrogels to adjust calvarial bone defects (Hayashi et al., 2009; Fujioka-Kobayashi et al., 2012; Bölgen et al., 2014; Charoenlarp et al., 2018; Ritz et al., 2018; Fang et al., 2019). Otherwise, *in situ* forming hydrogels were directly injected into the defect area, molding the defect site (Ding et al., 2019; Shoji et al., 2020), thereby ensuring a tight interface with the surrounding bone (El-Sherbiny and Yacoub, 2013; Maisani et al., 2018). Microbeads can be also easily adapted to a complex bone defect. Injectable hydrogels or microbeads allow minimal invasion of surrounding tissues during delivery (Ding et al., 2019).

Hydrogels also are good candidates to incorporate cells or growth factors and act as a delivery system. Pullulan and/or dextran-based materials display unique properties in the field of bone regeneration compared with commonly used bone substitutes. They exhibit resorption ability (Miyahara et al., 2012; Bölgen et al., 2014; Frasca et al., 2017), thereby ensuring the gradual replacement with newly formed bone compared with HA/TCP ceramics that exhibit extensive *in situ* resorption latencies (Keller et al., 2012). Another interesting property of these polymers is their radiotransparency (Hayashi et al., 2009; Frasca et al., 2017; Fricain et al., 2018) meaning that it allows to follow radiologically the new bone formation.

Several studies investigated the osteogenic properties of these polysaccharides-derived biomaterials through the adjunction of mineral contents. Most of these studies show a significant improvement of bone regeneration by incorporation of mineral contents. HA supplementation was used in eight of these 10 studies. This stimulates bone tissue formation and gives osteoconductive properties to the biomaterial. In addition, dextran and/or pullulan have been reinforced with mineral materials to overcome their weak physical properties (Fricain et al., 2013; Schlaubitz et al., 2014; Takahata et al., 2015; Ribot et al., 2017; Fricain et al., 2018; Ding et al., 2019; Fang et al., 2019; Popescu et al., 2020; Maurel et al., 2021; Wang et al., 2021) and then improve their mechanical strength.

Another reported strategy to enhance bone regeneration of dextran and/or pullulan-based materials was the encapsulation of growth factors. Growth factors are widely used in BTE to provide more regulating cues to target cell proliferation and differentiation and effective bone repair (Tang et al., 2021). A total of 13 studies reported the ability of pullulan-based materials and dextran-based materials to deliver growth factors. Most of them (i.e., 11 studies) focused on BMP-2 which play critical roles in bone regeneration process (Rao et al., 2018; Tang et al., 2021). A sustained release of this growth factor appeared to be difficult as heterotopic bone formation is usually observed due to its overexpression. Customization of biomaterials could be an alternative to regulate BMP-2 delivery. For example, chemical modification of polysaccharides was performed to enhance growth factors bearing within the scaffold to act as a drug delivery system. The addition of carboxylate, benzylamide, and sulfated groups to dextran-based scaffold enabled to mimic heparin-like compounds (Lafont et al., 2004; Maire et al., 2005a; Degat et al., 2009), which is one of the ECM components that has high affinity with growth factors. Functionalized dextran thus exhibits binding capacity to heparin-binding growth factors, such as transforming growth factor-b1 (TGF-b1) (Maire et al., 2005b). As TGF-b1 and BMP-2 belong to the same superfamily and share one third of structural homology, functionalized dextran has the ability to bind to BMP-2 through its heparin-binding site (Maire et al., 2005a). This could be an alternative to the currently used collagen sponges that are approved by FDA to deliver BMP-2 (Maire et al., 2005a). Interestingly, heterotopic bone formation of BMP-2 loaded with heparin microparticles was investigated at high and low concentrations (0.12 and 0.01 mg/kg body weight) in a rat femoral defect (Hettiaratchi et al., 2020; Vantucci et al., 2021). BMP-2 delivery alone led to heterotopic bone formation whatever the concentration used. But when it was combined to heparin microparticles at a high dosage (30 µg per graft), a sustained release was observed and seemed to regulate bone formation.

Interestingly, no study reported the use of a pullulan/dextran-based material to deliver growth factor. This could be explained by the ability of these matrices to retain local growth factors (Fricain et al., 2013). The combination of these two polysaccharides might be an ideal candidate to provide a growth factor-free biomaterial for BTE applications.

Finally, three studies investigated dextran or pullulan/dextran-based hydrogels capability to incorporate progenitor cells to promote bone healing (Bölgen et al., 2014; Frasca et al., 2017; Ritz et al., 2018). Two approaches can be evidenced in such scaffolds. In one hand, cells entrapped into the scaffold can migrate at the defect site to initiate the ossification process. On the other hand, they can stay inside the scaffold to recruit local factors. In these three studies, only one mentioned the use of pullulan/dextran biomaterial as a cell delivery system (Frasca et al., 2017). The two others did not specify the role of their biomaterial on the delivery of cells. Concerning the angiogenesis process of these biomaterials in bone defects, it was observed in all studies. However, no significant difference in bone regeneration amount was observed between the seeded scaffold and the cell-free scaffold in two studies, thereby suggesting that using dextran-based material as a potential cell carrier system does not increase its osteogenic potential. This could be related to a low ability of these polysaccharide-based hydrogels to support cell adhesion and proliferation. To overcome this drawback, Frasca et al. (2017) proposed a sequential multiple MSC administration strategy to cover the entirety of the repair process kinetic.

Only three studies (Takahata et al., 2015; Frasca et al., 2017; Fricain et al., 2018) compared the pullulan and pullulan/dextran-based materials to commercialized and commonly used bone substitutes. These commercial devices were either xenograft (e.g., Bio-Oss®, extract from the mineral part of bovine bone) or alloplastic bone substitutes (e.g., Calciresorb C35®, composed of HA and TCP and Biopex-R®, composed of a-TCP). Pullulan and pullulan/dextran-based materials seemed to be at least as efficient as these conventional and commercially available bone substitutes. Besides, alloplastic bone substitutes showed poor host integration and failed to completely resorb *in vivo,* thereby preventing their replacement by newly formed bone. However, further studies comparing these polysaccharides to commercially used bone substitutes should be conducted to draw formal conclusions.

Finally, there are limitations related to the present systematic review that must be mentioned. One limitation of this study is the heterogeneity in animal models and the wide types of bone defects performed in the included studies, thereby making it difficult to compare studies. Calvarial and femoral defects were the most commonly used model to assess bone regeneration in these studies. Femoral defect may be more appropriate to consider load-bearing capacity of polysaccharide scaffolds when necessary (Taguchi and Lopez, 2021). Another identified drawback of this systematic review is the low number of studies investigating the mechanical properties of the materials (Abbah et al., 2012; Takahata et al., 2015; Ding et al., 2019; Fang et al., 2019). Among four studies, only one study evaluated the compressive strength directly on the bone with the investigating constructs (Abbah et al., 2012). It would be interesting to further evaluate the mechanical properties of these constructs and their evolution in time that should match the natural bone properties (Yunus Basha et al., 2015) to favor host bone integration (Giannoudis et al., 2007). Another interesting parameter to consider when evaluating a biomaterial for bone regeneration is the defect size, as non-critical size defect may heal spontaneously. Originally, critical-sized defect can be defined as the smallest size tissue defect that will not completely heal over the natural lifetime of an animal (Schmitz and Hollinger, 1986; Spicer et al., 2012). In practice, a defect is considered as critical-sized defect if it does not fully heal during the experimental time observation (Gosain et al., 2000; McGovern et al., 2018). Among the 28 included studies, all experiments using calvarial defects could be considered as critical-sized defect [e.g., more than 2 mm in mouse; more than 5 mm in rats (Taguchi and Lopez, 2021)]. For other bone models (e.g., tibia, ulnar, vertebrae), knowledge on the size considered as critical defect in those models could not be found in the literature. It would thus be interesting to compare these polysaccharides using the same animal model. The lack of empty defect condition as control in 13 studies (Lafont et al., 2004; Chen et al., 2005; Chen et al., 2007; Fujioka-Kobayashi et al., 2012; Bölgen et al., 2014; Togami et al., 2015; Ribot et al., 2017; Charoenlarp et al., 2018; Fricain et al., 2018; Ding et al., 2019; Fang et al., 2019; Popescu et al., 2020; Maurel et al., 2021), prevented readers to conclude on the relevance of the model used and the tested scaffolds. Further studies including an empty defect condition in the animal experimental design would be necessary to increase the relevance of results. We also observed that none of the included studies performed the required sample size calculation for each condition before conducting the experiments. It would be useful to report these data in future studies to better understand the study design. In addition, only two studies conducted a blinded assessment of outcomes. Blind assessment of the collected data reduces the risk of bias when interpreting the data. If possible, this methodological parameter should be implemented in future preclinical studies to strengthen the level of evidence.

## 5 Conclusion

The present study emphasized that pullulan and/or dextran-based materials display unique properties for BTE applications. Moreover, this review is expected to provide clear information on the chemical functionalization of the pullulan-based and dextran-based materials and the cross-linking strategies for BTE applications. Interestingly, these polysaccharides are used as injectable hydrogels or microbeads that easily fit the bone defect. Incorporation of mineral contents such as hydroxyapatite to the pullulan and/or dextran-based biomaterials significantly enhance bone regeneration. We also outlined that the association of pullulan and dextran is a solution to obtain a biomaterial, deprived of growth factors or living cells to promote bone formation. Studies comparing these biomaterials with commercialized and clinically used products are too limited and further studies are required to draw more conclusions.

## References

[B1] Abbah S.-A. Liu J. Lam R. W. M. Goh J. C. H. Wong H.-K. (2012). *In Vivo* bioactivity of rhBMP-2 Delivered with Novel Polyelectrolyte Complexation Shells Assembled on an Alginate Microbead Core Template. J. Control. Release 162, 364–372. 10.1016/j.jconrel.2012.07.027 22846985

[B2] Akiyoshi K. Kobayashi S. Shichibe S. Mix D. Baudys M. Wan Kim S. (1998). Self-assembled Hydrogel Nanoparticle of Cholesterol-Bearing Pullulan as a Carrier of Protein Drugs: Complexation and Stabilization of Insulin. J. Control. Release 54, 313–320. 10.1016/s0168-3659(98)00017-0 9766251

[B3] Ana I. D. Satria G. A. P. Dewi A. H. Ardhani R. (2018). Bioceramics for Clinical Application in Regenerative Dentistry. Adv. Exp. Med. Biol. 1077, 309–316. 10.1007/978-981-13-0947-2_16 30357695

[B4] Banerjee A. Bandopadhyay R. (2016). Use of Dextran Nanoparticle: A Paradigm Shift in Bacterial Exopolysaccharide Based Biomedical Applications. Int. J. Biol. Macromol. 87, 295–301. 10.1016/j.ijbiomac.2016.02.059 26927936

[B5] Barritault D. Gilbert-Sirieix M. Rice K. L. Siñeriz F. Papy-Garcia D. Baudouin C. (2017). RGTA or ReGeneraTing Agents Mimic Heparan Sulfate in Regenerative Medicine: from Concept to Curing Patients. Glycoconj. J. 34, 325–338. 10.1007/s10719-016-9744-5 27924424 PMC5487810

[B6] Bölgen N. Korkusuz P. Vargel İ. Kılıç E. Güzel E. Çavuşoğlu T. (2014). Stem Cell Suspension Injected HEMA-Lactate-Dextran Cryogels for Regeneration of Critical Sized Bone Defects. Artif. Cells, Nanomedicine, Biotechnol. 42, 70–77. 10.3109/21691401.2013.775578 23477355

[B7] Charoenlarp P. Rajendran A. K. Fujihara R. Kojima T. Nakahama K.-i. Sasaki Y. (2018). The Improvement of Calvarial Bone Healing by Durable Nanogel-Crosslinked Materials. J. Biomaterials Sci. Polym. Ed. 29, 1876–1894. 10.1080/09205063.2018.1517403 30156966

[B8] Chen F.-m. Zhao Y.-m. Wu H. Deng Z.-h. Wang Q.-t. Zhou W. (2006). Enhancement of Periodontal Tissue Regeneration by Locally Controlled Delivery of Insulin-like Growth Factor-I from Dextran-Co-Gelatin Microspheres. J. Control. Release 114, 209–222. 10.1016/j.jconrel.2006.05.014 16859799

[B9] Chen F.-M. Zhao Y.-M. Zhang R. Jin T. Sun H.-H. Wu Z.-F. (2007). Periodontal Regeneration Using Novel Glycidyl Methacrylated Dextran (Dex-GMA)/gelatin Scaffolds Containing Microspheres Loaded with Bone Morphogenetic Proteins. J. Control. Release 121, 81–90. 10.1016/j.jconrel.2007.05.023 17617489

[B10] Chen F. Wu Z. Wang Q. Wu H. Zhang Y. Nie X. (2005). Preparation and Biological Characteristics of Recombinant Human Bone Morphogenetic Protein-2-Loaded Dextran-Co-Gelatin Hydrogel Microspheres, *In Vitro* and *In Vivo* Studies. Pharmacology 75, 133–144. 10.1159/000088212 16155372

[B11] Chen H. Yu Y. Wang C. Wang J. Liu C. (2019). The Regulatory Role of Sulfated Polysaccharides in Facilitating rhBMP-2-Induced Osteogenesis. Biomater. Sci. 7, 4375–4387. 10.1039/c9bm00529c 31429425

[B12] Degat M.-C. Dubreucq G. Meunier A. Dahri-Correia L. Sedel L. Petite H. (2009). Enhancement of the Biological Activity of BMP-2 by Synthetic Dextran Derivatives. J. Biomed. Mat. Res. 88A, 174–183. 10.1002/jbm.a.31884 18286621

[B13] Dimitriou R. Jones E. McGonagle D. Giannoudis P. V. (2011). Bone Regeneration: Current Concepts and Future Directions. BMC Med. 9, 66. 10.1186/1741-7015-9-66 21627784 PMC3123714

[B14] Ding X. Li X. Li C. Qi M. Zhang Z. Sun X. (2019). Chitosan/Dextran Hydrogel Constructs Containing Strontium-Doped Hydroxyapatite with Enhanced Osteogenic Potential in Rat Cranium. ACS Biomater. Sci. Eng. 5, 4574–4586. 10.1021/acsbiomaterials.9b00584 33448831

[B15] El-Sherbiny I. M. Yacoub M. H. (2013). Hydrogel Scaffolds for Tissue Engineering: Progress and Challenges. Glob. Cardiol. Sci. Pract. 2013, 38–342. 10.5339/gcsp.2013.38 PMC3963751 24689032

[B16] Fang J. Li P. Lu X. Fang L. Lü X. Ren F. (2019). A Strong, Tough, and Osteoconductive Hydroxyapatite Mineralized Polyacrylamide/dextran Hydrogel for Bone Tissue Regeneration. Acta Biomater. 88, 503–513. 10.1016/j.actbio.2019.02.019 30772515

[B17] Filippi M. Born G. Chaaban M. Scherberich A. (2020). Natural Polymeric Scaffolds in Bone Regeneration. Front. Bioeng. Biotechnol. 8, 474. 10.3389/fbioe.2020.00474 32509754 PMC7253672

[B18] Frasca S. Norol F. Le Visage C. Collombet J.-M. Letourneur D. Holy X. (2017). Calcium-phosphate Ceramics and Polysaccharide-Based Hydrogel Scaffolds Combined with Mesenchymal Stem Cell Differently Support Bone Repair in Rats. J. Mater Sci. Mater Med. 28, 35. 10.1007/s10856-016-5839-6 28110459 PMC5253158

[B19] Fricain J. C. Aid R. Lanouar S. Maurel D. B. Le Nihouannen D. Delmond S. (2018). In-vitro and Iin-Vvivo Design and Validation of an Injectable Polysaccharide-Hydroxyapatite Composite Material for Sinus Floor Augmentation. Dent. Mater. 34, 1024–1035. 10.1016/j.dental.2018.03.021 29636238

[B20] Fricain J. C. Schlaubitz S. Le Visage C. Arnault I. Derkaoui S. M. Siadous R. (2013). A Nano-Hydroxyapatite - Pullulan/dextran Polysaccharide Composite Macroporous Material for Bone Tissue Engineering. Biomaterials 34, 2947–2959. 10.1016/j.biomaterials.2013.01.049 23375393

[B21] Fujioka-Kobayashi M. Ota M. S. Shimoda A. Nakahama K.-i. Akiyoshi K. Miyamoto Y. (2012). Cholesteryl Group- and Acryloyl Group-Bearing Pullulan Nanogel to Deliver BMP2 and FGF18 for Bone Tissue Engineering. Biomaterials 33, 7613–7620. 10.1016/j.biomaterials.2012.06.075 22800537

[B22] Fundueanu G. Constantin M. Mihai D. Bortolotti F. Cortesi R. Ascenzi P. (2003). Pullulan-cyclodextrin Microspheres. J. Chromatogr. B 791, 407–419. 10.1016/s1570-0232(03)00258-7 12798201

[B23] Giannoudis P. V. Einhorn T. A. Marsh D. (2007). Fracture Healing: the Diamond Concept. Injury 38 (Suppl. 4), S3–S6. 10.1016/s0020-1383(08)70003-2 18224731

[B24] Gliko-Kabir I. Yagen B. Penhasi A. Rubinstein A. (2000). Phosphated Crosslinked Guar for Colon-specific Drug Delivery. J. Control. Release 63, 121–127. 10.1016/S0168-3659(99)00179-0 10640585

[B25] Gosain A. K. Song L. Yu P. Mehrara B. J. Maeda C. Y. Gold L. I. (2000). Osteogenesis in Cranial Defects: Reassessment of the Concept of Critical Size and the Expression of TGF-β Isoforms. Plastic Reconstr. Surg. 106, 360–371. 10.1097/00006534-200008000-00018 10946935

[B26] Hayashi C. Hasegawa U. Saita Y. Hemmi H. Hayata T. Nakashima K. (2009). Osteoblastic Bone Formation Is Induced by Using Nanogel-Crosslinking Hydrogel as Novel Scaffold for Bone Growth Factor. J. Cell. Physiol. 220, 1–7. 10.1002/jcp.21760 19301257

[B27] Hettiaratchi M. H. Krishnan L. Rouse T. Chou C. McDevitt T. C. Guldberg R. E. (2020). Heparin-mediated Delivery of Bone Morphogenetic Protein-2 Improves Spatial Localization of Bone Regeneration. Sci. Adv. 6, eaay1240. 10.1126/sciadv.aay1240 31922007 PMC6941907

[B28] Hooijmans C. R. Rovers M. M. de Vries R. B. Leenaars M. Ritskes-Hoitinga M. Langendam M. W. (2014). SYRCLE's Risk of Bias Tool for Animal Studies. BMC Med. Res. Methodol. 14, 43. 10.1186/1471-2288-14-43 24667063 PMC4230647

[B29] Hovgaard L. Brøndsted H. (1995). Dextran Hydrogels for Colon-specific Drug Delivery. J. Control. Release 36, 159–166. 10.1016/0168-3659(95)00049-E

[B30] Hussain A. Zia K. M. Tabasum S. Noreen A. Ali M. Iqbal R. (2017). Blends and Composites of Exopolysaccharides; Properties and Applications: A Review. Int. J. Biol. Macromol. 94, 10–27. 10.1016/j.ijbiomac.2016.09.104 27697492

[B31] Kashirina A. Yao Y. Liu Y. Leng J. (2019). Biopolymers as Bone Substitutes: a Review. Biomater. Sci. 7, 3961–3983. 10.1039/c9bm00664h 31364613

[B32] Kato N. Hasegawa U. Morimoto N. Saita Y. Nakashima K. Ezura Y. (2007). Nanogel-based Delivery System Enhances PGE2 Effects on Bone Formation. J. Cell. Biochem. 101, 1063–1070. 10.1002/jcb.21160 17520665

[B33] Keller J. Brink S. Busse B. Schilling A. F. Schinke T. Amling M. (2012). Divergent Resorbability and Effects on Osteoclast Formation of Commonly Used Bone Substitutes in a Human In Vitro-assay. PloS One 7, e46757. 10.1371/journal.pone.0046757 23071629 PMC3468634

[B34] Krishnakumar G. S. Sampath S. Muthusamy S. John M. A. (2019). Importance of Crosslinking Strategies in Designing Smart Biomaterials for Bone Tissue Engineering: A Systematic Review. Mater. Sci. Eng. C 96, 941–954. 10.1016/j.msec.2018.11.081 30606606

[B35] Kumari L. Badwaik H. R. (2019). “Polysaccharide-based Nanogels for Drug and Gene Delivery,” in Polysaccharide Carriers for Drug Delivery. Editors Maiti S. Jana S. (Sawston, United Kingdom: Woodhead Publishing), 497–557. 10.1016/B978-0-08-102553-6.00018-0

[B36] Lack S. Dulong V. Picton L. Cerf D. L. Condamine E. (2007). High-resolution Nuclear Magnetic Resonance Spectroscopy Studies of Polysaccharides Crosslinked by Sodium Trimetaphosphate: a Proposal for the Reaction Mechanism. Carbohydr. Res. 342, 943–953. 10.1016/j.carres.2007.01.011 17303095

[B37] Lafont J. Blanquaert F. Colombier M. L. Barritault D. Carueelle J.-P. Saffar J.-L. (2004). Kinetic Study of Early Regenerative Effects of RGTA11, a Heparan Sulfate Mimetic, in Rat Craniotomy Defects. Calcif. Tissue Int. 75, 517–525. 10.1007/s00223-004-0012-5 15654496

[B38] Lalande C. Miraux S. Miraux S. Derkaoui S. Mornet S. Bareille R. (2011). Magnetic Resonance Imaging Tracking of Human Adipose Derived Stromal Cells within Three-Dimensional Scaffolds for Bone Tissue Engineering. eCM 21, 341–354. 10.22203/ecm.v021a25 21484704

[B40] Li X. Yang Z. Fang L. Ma C. Zhao Y. Liu H. (2021). Hydrogel Composites with Different Dimensional Nanoparticles for Bone Regeneration. Macromol. Rapid Commun. 42, 2100362. 10.1002/marc.202100362 34435714

[B41] Liang W. Wu X. Dong Y. Shao R. Chen X. Zhou P. (2021). *In Vivo* behavior of Bioactive Glass-Based Composites in Animal Models for Bone Regeneration. Biomater. Sci. 9, 1924–1944. 10.1039/d0bm01663b 33506819

[B42] Macleod M. R. O’Collins T. Howells D. W. Donnan G. A. (2004). Pooling of Animal Experimental Data Reveals Influence of Study Design and Publication Bias. Stroke 35, 1203–1208. 10.1161/01.STR.0000125719.25853.20 15060322

[B43] Maire M. Chaubet F. Mary P. Blanchat C. Meunier A. Logeartavramoglou D. (2005a). Bovine BMP Osteoinductive Potential Enhanced by Functionalized Dextran-Derived Hydrogels. Biomaterials 26, 5085–5092. 10.1016/j.biomaterials.2005.01.020 15769544

[B44] Maire M. Logeart-Avramoglou D. Degat M.-C. Chaubet F. (2005b). Retention of Transforming Growth Factor β1 Using Functionalized Dextran-Based Hydrogels. Biomaterials 26, 1771–1780. 10.1016/j.biomaterials.2004.06.003 15576151

[B45] Maisani M. Sindhu K. R. Fenelon M. Siadous R. Rey S. Mantovani D. (2018). Prolonged Delivery of BMP-2 by a Non-polymer Hydrogel for Bone Defect Regeneration. Drug Deliv. Transl. Res. 8, 178–190. 10.1007/s13346-017-0451-y 29192408

[B46] Mallick K. K. Cox S. C. (2013). Biomaterial Scaffolds for Tissue Engineering. Front. Biosci. 5, 341–360. 10.2741/e620 23276994

[B47] Maurel D. B. Fénelon M. Aid‐Launais R. Bidault L. Le Nir A. Renard M. (2021). Bone Regeneration in Both Small and Large Preclinical Bone Defect Models Using an Injectable Polymer‐based Substitute Containing Hydroxyapatite and Reconstituted with Saline or Autologous Blood. J. Biomed. Mater Res. 109, 1840–1848. 10.1002/jbm.a.37176 33797182

[B48] McGovern J. A. Griffin M. Hutmacher D. W. (2018). Animal Models for Bone Tissue Engineering and Modelling Disease. Dis. Model. Mech. 11, dmm033084. 10.1242/dmm.033084 29685995 PMC5963860

[B49] Miyahara T. Nyan M. Shimoda A. Yamamoto Y. Kuroda S. Shiota M. (2012). Exploitation of a Novel Polysaccharide Nanogel Cross-Linking Membrane for Guided Bone Regeneration (GBR). J. Tissue Eng. Regen. Med. 6, 666–672. 10.1002/term.475 22095663

[B50] Moher D. Shamseer L. Shamseer L. Clarke M. Ghersi D. Liberati A. (2015). Preferred Reporting Items for Systematic Review and Meta-Analysis Protocols (PRISMA-P) 2015 Statement. Syst. Rev. 4, 1. 10.1186/2046-4053-4-1 25554246 PMC4320440

[B51] Morimoto N. Endo T. Iwasaki Y. Akiyoshi K. (2005a). Design of Hybrid Hydrogels with Self-Assembled Nanogels as Cross-Linkers: Interaction with Proteins and Chaperone-like Activity. Biomacromolecules 6, 1829–1834. 10.1021/bm050156x 16004415

[B52] Morimoto N. Endo T. Ohtomi M. Iwasaki Y. Akiyoshi K. (2005b). Hybrid Nanogels with Physical and Chemical Cross-Linking Structures as Nanocarriers. Macromol. Biosci. 5, 710–716. 10.1002/mabi.200500051 16080166

[B53] Oryan A. Kamali A. Moshiri A. Baharvand H. Daemi H. (2018). Chemical Crosslinking of Biopolymeric Scaffolds: Current Knowledge and Future Directions of Crosslinked Engineered Bone Scaffolds. Int. J. Biol. Macromol. 107, 678–688. 10.1016/j.ijbiomac.2017.08.184 28919526

[B54] Popescu R. A. Tăbăran F. A. Bogdan S. Fărcăṣanu A. Purdoiu R. Magyari K. (2020). Bone Regeneration Response in an Experimental Long Bone Defect Orthotopically Implanted with Alginate‐pullulan‐glass‐ceramic Composite Scaffolds. J. Biomed. Mater Res. 108, 1129–1140. 10.1002/jbm.b.34464 31397056

[B55] Prajapati V. D. Jani G. K. Khanda S. M. (2013). Pullulan: an Exopolysaccharide and its Various Applications. Carbohydr. Polym. 95, 540–549. 10.1016/j.carbpol.2013.02.082 23618305

[B39] Lanza R. Langer R. Vacanti J. (Editors) (2014). Principles of Tissue Engineering. 4th ed. (Elsevier). 10.1016/C2011-0-07193-4

[B56] Rao S. H. Harini B. Shadamarshan R. P. K. Balagangadharan K. Selvamurugan N. (2018). Natural and Synthetic Polymers/bioceramics/bioactive Compounds-Mediated Cell Signalling in Bone Tissue Engineering. Int. J. Biol. Macromol. 110, 88–96. 10.1016/j.ijbiomac.2017.09.029 28917940

[B57] Ribot E. J. Tournier C. Aid-Launais R. Koonjoo N. Oliveira H. Trotier A. J. (2017). 3D Anatomical and Perfusion MRI for Longitudinal Evaluation of Biomaterials for Bone Regeneration of Femoral Bone Defect in Rats. Sci. Rep. 7, 6100. 10.1038/s41598-017-06258-0 28733632 PMC5522444

[B58] Ritz U. Eberhardt M. Klein A. Frank P. Götz H. Hofmann A. (2018). Photocrosslinked Dextran-Based Hydrogels as Carrier System for the Cells and Cytokines Induce Bone Regeneration in Critical Size Defects in Mice. Gels 4, 63. 10.3390/gels4030063 PMC6209263 30674839

[B59] Roseti L. Parisi V. Petretta M. Cavallo C. Desando G. Bartolotti I. (2017). Scaffolds for Bone Tissue Engineering: State of the Art and New Perspectives. Mater. Sci. Eng. C 78, 1246–1262. 10.1016/j.msec.2017.05.017 28575964

[B60] Schlaubitz S. Derkaoui S. M. Marosa L. Miraux S. Renard M. Catros S. (2014). Pullulan/dextran/nHA Macroporous Composite Beads for Bone Repair in a Femoral Condyle Defect in Rats. PloS One 9, e110251. 10.1371/journal.pone.0110251 25330002 PMC4203774

[B61] Schmitz J. P. Hollinger J. O. (1986). The Critical Size Defect as an Experimental Model for Craniomandibulofacial Nonunions. Clin. Orthop. Relat. Res. 205, 299–308. 10.1097/00003086-198604000-00036 3084153

[B62] Shoji S. Uchida K. Saito W. Sekiguchi H. Inoue G. Miyagi M. (2020). Acceleration of Bone Healing by In Situ-Forming Dextran-Tyramine Conjugates Containing Basic Fibroblast Growth Factor in Mice. Cureus 12, e10085. 10.7759/cureus.10085 32874816 PMC7455394

[B63] Simonsen L. Hovgaard L. Mortensen P. B. Brøndsted H. (1995). Dextran Hydrogels for Colon-specific Drug Delivery. V. Degradation in Human Intestinal Incubation Models. Eur. J. Pharm. Sci. 3, 329–337. 10.1016/0928-0987(95)00023-6

[B64] Spicer P. P. Kretlow J. D. Young S. Jansen J. A. Kasper F. K. Mikos A. G. (2012). Evaluation of Bone Regeneration Using the Rat Critical Size Calvarial Defect. Nat. Protoc. 7, 1918–1929. 10.1038/nprot.2012.113 23018195 PMC3513397

[B65] Sun G. Zhang X. Shen Y.-I. Sebastian R. Dickinson L. E. Fox-Talbot K. (2011). Dextran Hydrogel Scaffolds Enhance Angiogenic Responses and Promote Complete Skin Regeneration during Burn Wound Healing. Proc. Natl. Acad. Sci. U.S.A. 108, 20976–20981. 10.1073/pnas.1115973108 22171002 PMC3248550

[B66] Taguchi T. Lopez M. J. (2021). An Overview of De Novo Bone Generation in Animal Models. J. Orthop. Res. 39, 7–21. 10.1002/jor.24852 32910496 PMC7820991

[B67] Takahata T. Okihara T. Yoshida Y. Yoshihara K. Shiozaki Y. Yoshida A. (2015). Bone Engineering by Phosphorylated-Pullulan and β -TCP Composite. Biomed. Mat. 10, 065009. 10.1088/1748-6041/10/6/065009 26586655

[B68] Tang G. Liu Z. Liu Y. Yu J. Wang X. Tan Z. (2021). Recent Trends in the Development of Bone Regenerative Biomaterials. Front. Cell Dev. Biol. 9, 665813. 10.3389/fcell.2021.665813 34026758 PMC8138062

[B69] Tassa C. Shaw S. Y. Weissleder R. (2011). Dextran-coated Iron Oxide Nanoparticles: a Versatile Platform for Targeted Molecular Imaging, Molecular Diagnostics, and Therapy. Acc. Chem. Res. 44, 842–852. 10.1021/ar200084x 21661727 PMC3182289

[B70] Togami W. Sei A. Okada T. Taniwaki T. Fujimoto T. Tahata S. (2015). Effects of the Water-Holding Capability of Polyvinyl Formal Sponges on Osteogenic Ability Inin Vivoexperiments. J. Biomed. Mat. Res. 103, 188–194. 10.1002/jbm.b.33200 24819983

[B71] Vantucci C. E. Krishan L. Cheng A. Prather A. Roy K. Guldberg R. E. (2021). BMP-2 Delivery Strategy Modulates Local Bone Regeneration and Systemic Immune Responses to Complex Extremity Trauma. Biomater. Sci. 9, 1668–1682. 10.1039/d0bm01728k 33409509 PMC8256799

[B72] Wang B. Liu J. Niu D. Wu N. Yun W. Wang W. (2021). Mussel-Inspired Bisphosphonated Injectable Nanocomposite Hydrogels with Adhesive, Self-Healing, and Osteogenic Properties for Bone Regeneration. ACS Appl. Mat. Interfaces 13, 32673–32689. 10.1021/acsami.1c06058 34227792

[B73] Wu D. T. Munguia-Lopez J. G. Cho Y. W. Ma X. Song V. Zhu Z. (2021). Polymeric Scaffolds for Dental, Oral, and Craniofacial Regenerative Medicine. Molecules 26, 7043. 10.3390/molecules26227043 34834134 PMC8621873

[B74] Yu Y. Chen R. Yuan Y. Wang J. Liu C. (2020). Affinity-selected Polysaccharide for rhBMP-2-Induced Osteogenesis via BMP Receptor Activation. Appl. Mater. Today 20, 100681. 10.1016/j.apmt.2020.100681

[B75] Yunus Basha R. T.S. S. K. Doble M. (2015). Design of Biocomposite Materials for Bone Tissue Regeneration. Mater. Sci. Eng. C 57, 452–463. 10.1016/j.msec.2015.07.016 26354284

